# Diel patterns in swimming behavior of a vertically migrating deepwater shark, the bluntnose sixgill (*Hexanchus griseus*)

**DOI:** 10.1371/journal.pone.0228253

**Published:** 2020-01-24

**Authors:** Daniel M. Coffey, Mark A. Royer, Carl G. Meyer, Kim N. Holland

**Affiliations:** Hawai‘i Institute of Marine Biology, University of Hawai‘i at Mānoa, Kāne‘ohe, Hawai‘i, United States of America; Institut de recherche pour le developpement, FRANCE

## Abstract

Diel vertical migration is a widespread behavioral phenomenon where organisms migrate through the water column and may modify behavior relative to changing environmental conditions based on physiological tolerances. Here, we combined a novel suite of biologging technologies to examine the thermal physiology (intramuscular temperature), fine-scale swimming behavior and activity (overall dynamic body acceleration as a proxy for energy expenditure) of bluntnose sixgill sharks (*Hexanchus griseus*) in response to environmental changes (depth, water temperature, dissolved oxygen) experienced during diel vertical migrations. In the subtropical waters off Hawai‘i, sixgill sharks undertook pronounced diel vertical migrations and spent considerable amounts of time in cold (5–7°C), low oxygen conditions (10–25% saturation) during their deeper daytime distribution. Further, sixgill sharks spent the majority of their deeper daytime distribution with intramuscular temperatures warmer than ambient water temperatures, thereby providing them with a significant thermal advantage over non-vertically migrating and smaller-sized prey. Sixgill sharks exhibited relatively high rates of activity during both shallow (night) and deep (day) phases and contrary to our predictions, did not reduce activity levels during their deeper daytime distribution while experiencing low temperature and dissolved oxygen levels. This demonstrates an ability to tolerate the low oxygen conditions occurring within the local oxygen minimum zone. The novel combination of biologging technologies used here enabled innovative *in situ* deep-sea natural experiments and provided significant insight into the behavioral and physiological ecology of an ecologically important deepwater species.

## Introduction

Diel vertical migration, where organisms migrate through the water column for feeding, predator/competitor avoidance or to maximize energetic efficiency, is a widespread phenomenon in aquatic ecosystems [1–5]. Based on physiological tolerances, organisms may alter their behavior in response to changing environmental conditions encountered during migration [6–9]. In the subtropical waters off Hawai‘i, bluntnose sixgill sharks (*Hexanchus griseus*; hereafter referred to as sixgill sharks) undergo a distinct diel vertical migration descending to depths below 500 m during the day and ascending to 200–350 m during the night and encounter a wide range of temperatures (5–20°C [10–11]) and dissolved oxygen conditions (9–85% saturation [12]; <60–200 μmol kg^-1^ [10]). Sixgill sharks appear to be highly tolerant of low dissolved oxygen, remaining in waters that are generally considered hypoxic (defined as <61 μmol kg^-1^ [13–15]) within the local oxygen minimum zone (OMZ) throughout their deeper, daytime distribution [10,12,16].

Diel vertical migrators may tolerate hypoxic conditions during daytime forays into OMZs via: (1) enhanced oxygen extraction and transport in the gills, blood and tissues; (2) upregulation of anaerobic metabolism; and (3) suppression of total energy consumption by reducing energetically-expensive processes such as locomotor activity [17,18]. Further, these are not mutually exclusive, and many organisms employ a combination of these strategies to accommodate local oxygen conditions and expand vertical habitat [17,18]. Therefore, sixgill sharks may restrict locomotor activity as a strategy to reduce overall metabolic expenditure in order to facilitate brief (hours) forays into cold, hypoxic water [14,17,18].

Ectothermic species, such as sixgill sharks, must also contend with changes in environmental temperature, which has a strong influence on physiology and fitness from individual to community scales [19–22]. Sixgill sharks off Hawai’i spend their shallower nighttime distribution (200–350 m) at variable temperatures (11–15°C) within the thermocline and the majority of their daytime distribution occupying stable, cold temperatures (5–7°C) at depths below 500 m [10,11]. As such, sixgill sharks may modify their behavior to enable them to take advantage of diverse thermal habitats [23,24]. For example, the large body mass of sixgill sharks may provide sufficient thermal inertia to slow heat loss during their descent into cold, deep waters [25,26]. Thermal inertia may enable sixgill sharks to maintain relatively high activity levels for longer periods at colder temperatures, and thus exploit deep prey resources more effectively compared to resident (non-vertically migrating) species. Sixgill sharks could also migrate between foraging and non-foraging habitats to select optimal temperatures for bioenergetic and/or digestive efficiency [23,27,28].

Recent advances in biologging have enabled the recording of *in situ* dissolved oxygen contemporaneously with the behavior of the tagged animal [12]. Here, we combined this novel technology with an accelerometer, temperature-depth recorder and intramuscular thermistor to examine for the first time the fine-scale swimming behavior, activity, and thermal physiology of sixgill sharks in response to environment changes experienced during diel vertical migrations. Specifically, we hypothesize that sixgill sharks reduce activity levels during their deeper daytime distribution in response to low temperature and dissolved oxygen conditions.

## Materials and methods

### Data collection

Five sixgill sharks (2 male, 3 female; Table 1) were captured using demersal longlines set off Kāne‘ohe Bay, O‘ahu, Hawai‘i (21.46°N, 157.80°W) (for additional details of longline fishing methods see [11]). Both males (326 and 352 cm total length [TL]) were mature and the three females (309, 346, and 412 cm TL) were large subadults based on size at maturity estimates [29]. To quantify swimming behavior, we used a TDR10-X accelerometer (57 × 38 × 24 mm, 69 g; Wildlife Computers, Redmond, WA, USA), which recorded tri-axial acceleration at 1/32-s intervals. Environmental data were obtained using a prototype dissolved oxygen pop-up satellite archival tag (DO-PAT: 170 × ⌀ 60 mm, 85 g; Wildlife Computers, Redmond, WA, USA; for additional details see [12]) or a prototype TDR10-DO inline finmount tag (109 × 53 × 21 mm, 118g; Wildlife Computers, Redmond, WA, USA) equipped with a micro dissolved oxygen probe (27 × ⌀ 12 mm; OxyGuard International A/S, Farum, Denmark). In total, this combination of sensors recorded depth, water temperature, and dissolved oxygen saturation at 1-s intervals. Intramuscular temperature was recorded at 10-s intervals using a TDR-Mk9 archival tag (71 × 17 × 17 mm, 120 mm stalk length, 29 g; Wildlife Computers, Redmond, WA, USA) with a temperature sensor incorporated on a sensor stalk that was inserted approximately 8 cm below the skin into the dorsal musculature. These devices were embedded in small, syntactic-foam floats equipped with an Argos satellite transmitter and UHF radio tracking beacon (MiniPAT: 115 × ⌀ 40 mm, 53 g; or SPOT-216E: 80 × 20 × 11 mm, 30 g; Wildlife Computers, Redmond, WA, USA), and a timed-release mechanism (19 × ⌀ 16 mm, 10 g; Little Leonardo Co., Tokyo, Japan). The floats provided just enough overall buoyancy to return the instrument package to the surface on release. Total masses of the devices (400 and 528 g in air) were less than 0.3% of the shark’s estimated mass (Table 1). To attach the package a hole was pierced on the central dorsal surface through the skin with a sharp probe and the device was secured parallel to the longitudinal axis of the body by a stainless-steel cable tie (360 mm, 8 g; Little Leonardo Co., Tokyo, Japan) passed through the hole. After ~96 h, a pre-programmed release timer severed the cable tie and detached the package; however, the TDR-Mk9 sensor stalk took an additional 0.5–55 h to pull out of the dorsal musculature before allowing the package to float to the surface where the satellite and UHF transmitters enabled us to locate and retrieve the devices. Procedures were approved by the ethics committee at the University of Hawai‘i (Institutional Animal Care and Use Committee Protocol #05–053).

**Table 1:** Summary of deployments on bluntnose sixgill sharks (*Hexanchus griseus*). Day corresponds to deep day phases and night corresponds to shallow night phases (dawn descent and dusk ascent segments of diel vertical migrations are excluded).

Shark ID	Sex	Total Length (cm)	Estimated Mass (kg)^a^	Deployment Duration (h)	Depth (m)			Water Temperature (°C)			% Oxygen Saturation		
					Day Median (IQR)	Night Median (IQR)	Range	Day Median (IQR)	Night Median (IQR)	Range	Day Median (IQR)	Night Median (IQR)	Range
HG1	M	352	247	112	588 (528–616)	267 (249–284)	180–684	6.2 (5.9–6.6)	13.4 (12.5–14.2)	4.9–16.0	16 (14–20)	73 (70–75)	11–89
HG2	F	412	408	110	580 (561–604)	350 (296–456)	228–667	6.2 (6.0–6.4)	10.4 (7.6–12.7)	5.3–15.6	19 (18–20)	40 (26–45)	15–56
HG3	M	326	195	151	614 (593–634)	277 (253–313)	182–693	5.7 (5.5–6.0)	13.6 (12.2–14.5)	5.1–17.3	19 (18–20)	72 (67–73)	16–86
HG4	F	309	166	112	566 (535–619)	284 (241–314)	185–699	5.8 (5.5–6.1)	11.9 (10.5–14.2)	4.9–17.7	20 (19–22)	72 (63–77)	13–80
HG5	F	346	234	96	545 (508–588)	296 (274–382)	231–816	6.8 (6.3–7.3)	13.4 (9.5–14.5)	4.7–16.0	23 (18–34)	78 (67–80)	10–86

IQR, interquartile range

^a^Estimated mass was calculated from the relationship between mass and total length for bluntnose sixgill sharks [30].

### Data processing

The first 15–40 h of each record were omitted from analysis because the behavior of the shark was inconsistent with the remainder of the record and indicated a recovery period after capture and handling. Analyses of acceleration and intramuscular temperature data concluded ~96 h post-deployment when the pre-programmed release timer severed the cable tie and detached the instrument package from a fixed position, which was evident in the acceleration data. The final 31 h of environmental data recorded by the DO-PAT from HG3 were corrupted, so only the first 120 h were analyzed. The accelerometer battery died after 10 h for HG4; therefore, only environmental and intramuscular temperature data were analyzed for this shark. After removing these data from the analysis, there was a total of 265 h (range 55.6–80.4 h) of accelerometer data from 4 sharks and 321.5 h (range 56.0–80.7 h) of intramuscular temperature and 402 h (range 56.1–105.0 h) of environmental data (depth, water temperature, and dissolved oxygen saturation) from 5 sharks.

Dissolved oxygen saturation data were smoothed relative to corresponding depth data using a robust local regression (LOESS) algorithm with a 5-s window in MATLAB (The MathWorks Inc., Natick, MA, USA) (sensu [12]). The smoothing algorithm effectively reduced the noise from the last deployment (HG5), but some outliers persisted for the remaining deployments. These outliers were identified and removed using studentized residuals and new values were based on nearest neighbor interpolation.

### Data analysis

#### Diel patterns

To describe overall diel patterns of habitat use, the proportion of time spent at depth (5 m bins), water temperature (0.5°C bins), and dissolved oxygen saturation (1% bins) by time of day was calculated at 15 min intervals for every complete 24 h period for each shark and then averaged across all sharks. Vertical displacement rates were calculated by dividing the change in depth between each record by the sampling rate. The majority (98%) of vertical displacement rates calculated using the high-resolution depth time series (recorded at 1-s intervals) ranged from 0–1 m s^-1^ (maximum 2.5 m s^-1^) with a depth sensor resolution of 0.5 m. Therefore, data were sub-sampled at 1-min intervals to better describe diel patterns in vertical displacement rates. The depth time series for each individual was visually examined and divided into four categories (sensu [31]): shallow night phase, deep day phase, and two crepuscular vertical migrations (dawn descent and dusk ascent). To describe patterns of habitat use for shallow night phases and deep day phases, the proportion of time spent at depth, water temperature, and dissolved oxygen saturation was calculated using the high-resolution time series for each shark and then averaged across all sharks.

#### Intramuscular temperature

To describe changes in intramuscular temperature across diel vertical migrations, we estimated changes in the whole-body heat-transfer coefficient *k* that are necessary to account for the observed rates of body warming and cooling using a function of heat exchange with the environment and internal (metabolic) heat production [7,32]. Heat loss (or gain) is proportional to the difference between the intramuscular temperature of the shark and ambient water temperatures:

$$\frac{dT_{b}(t)}{dt}=k(T_{a}(t)-T_{b}(t))+{\dot{T}}_{m}$$

where *k* is the whole-body heat transfer coefficient (°C min^-1^°C^-1^), *T_a_*(*t*) is the ambient water temperature (°C) as a function of time *t*, *T_b_*(*t*) is the intramuscular temperature (°C) of the shark as a function of time *t*, and ${\dot{T}}_{m}$ is the rate of temperature change due to internal (metabolic) heat production (°C min^-1^). The following two conditions of *k* were assumed:

1. *k* = a constant value;
2. $k=\left\{ \begin{array}{c} k_{1};\;Ta(t)<T_{b}(t)\\ k_{2};\;Ta(t)\geq T_{b}(t) \end{array}\right.$,

where *k*_1_ (cooling) and *k*_2_ (heating) are two values for the heat-transfer coefficient. The optimized parameters for each model were estimated based on minimization of the sum of squared errors (SSE).

Diel patterns in the proportion of time spent at intramuscular temperature (0.5°C bins) by time of day and for shallow night and deep day phases were calculated as described in the previous section. We measured the difference in intramuscular temperature from ambient water temperature as Δ*T* and calculated the proportion of time spent at Δ*T* (0.5°C bins) during shallow night and deep day phases for each shark and then averaged across all sharks.

#### Acceleration data

Acceleration data were analyzed using Igor Pro 6.3 (WaveMetrics Inc., Portland, OR, USA) with the ‘Ethographer’ package [33]. Raw acceleration data include both low-frequency gravity components (caused by the shark’s changing body inclination), and high-frequency specific components caused by dynamic movements such as tail beating. We separated specific and gravity components of acceleration using a 0.01 Hz low-pass filter, which removes high frequency waves to reveal the gravity component. We used the gravity component of acceleration to calculate the shark’s body inclination (pitch and roll; sensu [34]). Differences in the pitch angle between the longitudinal axis of the shark’s body and accelerometer were corrected using the relationship with vertical displacement rate as described in [35]. Acceleration data were categorized into three swimming phases (ascents, descents, level) based on pitch angles following [36], assuming pitch angles above 5° to be ascents, below -5° to be descents, and intermediate angles to be level swimming. We used continuous wavelet transformation to generate a spectrogram of swaying acceleration, classified the dominant peak as tail beat cycles, and calculated tail beat frequency and amplitude of acceleration at every second. Overall dynamic body acceleration (ODBA) was calculated by summing the absolute specific components of acceleration across all three orthogonal axes. Average (per minute) metrics of pitch and acceleration (tail beat frequency, amplitude of acceleration, and ODBA) during diel depth phases were compared for each individual.

We used generalized additive mixed models (GAMMs) to determine which temporal, physiological, and environmental factors influence ODBA of sixgill sharks. ODBA was used as a measure of activity (as opposed to tail beat frequency) since it measures change in behavior in all axes and has been used as a proxy for energy expenditure across taxa, including sharks [37–40]. We incorporated time of day, depth, water temperature, intramuscular temperature, dissolved oxygen saturation, and swimming phase (ascent, descent, level) as candidate predictor variables. For GAMM analyses, we sub-sampled data over 1-min means. Prior to model fitting, data exploration was carried out per [41]. Collinearity between candidate predictor variables was assessed with Pearson correlation coefficients and variance inflation factors (VIF) using the ‘corvif ‘ function [42] in R [43]. High absolute Pearson correlation coefficients (>0.73) and VIF (>3) indicated high collinearity between all predictor variables except for time of day and swimming phase. Therefore, a principal component analysis (PCA) was conducted on the correlated predictor variables in R. Principal components with greater than 10% of variance explained were included with time of day and swimming phase as candidate predictor variables in GAMM analyses. Time of day and swimming phase were also included with each of the correlated predictor variables in separate models to determine individual environmental effects on ODBA. Interactions between smooth terms were examined using tensor product smooths (t2; [44]). As observations were repeated measures collected from the same individuals, we modeled individual sharks as a random effect. We included a correlation structure with an autoregressive process of order 1 (AR1) to account for serial correlation in time series data [42]. ODBA was modeled using a Gaussian distribution with a log link function. Thin plate regression splines were estimated for each physiological and environmental variable whereas time of day was modeled using a cyclic cubic regression spline, which constrains the start and end points of the smooth term to be the same [45]. GAMMs were constructed in R using the ‘mgcv’ package [45] and model fits were compared using Akaike Information Criterion corrected for small sample size (AIC_c_) and Akaike weights (*w*) [46], calculation of percent deviance explained, and residual diagnostic plots. Models with substantial support were selected based on a ΔAIC_c_ < 2 from the model with the lowest AIC_c_.

We used Hidden Markov models (HMMs) to distinguish between different activity states based on ODBA. HMMs connect observed behavior data, such as ODBA, to an underlying latent process, generally interpreted as the animal’s unobserved behavior [47,48]. HMMs directly account for the serial dependence in our dataset [48,49] and allow modeling with a higher sampling resolution (i.e., seconds instead of minutes). In particular, HMMs provide a measure of the probability of sharks switching or persisting within behavioral states [48] and how these are driven by temporal, physiological, and/or environmental conditions. We applied a two-state HMM with one state post hoc interpreted as representing less active behavior and the other relatively more active behavior following [48] and [50]. For HMM analyses, we sub-sampled data over 10-s means corresponding to the longest sampling rate (10 s) of intramuscular temperature data. Mean ODBA values calculated over a period of 10 s capture 1–2 dominant tail beat cycles for sixgill sharks [11], thereby reducing the variability in activity estimates due to peaks and troughs in acceleration data during the tail beat cycle [51,52].

The state-dependent probability distributions were estimated using a gamma distribution. We considered several sets of initial values for the parameters of the state-dependent distributions and as a result are confident that we found the global optimum of the likelihood function. To examine potential temporal, physiological, and environmental effects on activity states, we allowed the entries of the transition probability matrix to be functions of up to three covariates including swimming phase, time of day, and either a correlated predictor variable or principal component (as described above) and their interaction. Time of day (*t*) is represented by two trigonometric functions with period 24 h, cos(2π*t*/8640) and sin(2π*t*/8640) (24-h periodicity is represented by 8640 time points since data were collected every 10 s). HMMs were constructed in R using the ‘momentuHMM’ package [53]. The ‘momentuHMM’ package currently does not have the capacity to include individual-level random effects; however, we performed a comparable analysis by including individual-level fixed effects on the state transition probabilities [53]. We calculated the HMM likelihood using the forward algorithm, which allows for parameter estimation via a numerical maximum likelihood approach [54]. Model fits were compared using AIC, Akaike weights, and pseudo-residuals [54]. Models with substantial support were selected based on a ΔAIC < 2 from the model with the lowest AIC. We used the Viterbi algorithm to decode the optimal state sequence underlying each time series, thus connecting each observation to one of the two states [54]. To determine how activity varied by depth, water temperature and dissolved oxygen saturation, we related the decoded state sequences to a grid of depth (5 m bins) and water temperature (0.5°C bins) cells and a grid of depth and dissolved oxygen saturation (5% bins) cells. For each grid cell, we calculated the percentage of decoded states corresponding to state 1 or state 2 following [48] and [50]. We estimated the stationary (equilibrium) state probabilities (i.e., the marginal probability of a state at a fixed value of the covariate; see [49]) as a function of each covariate included in the best-fit model. As sixgill sharks exhibit a distinct diel shift in habitat use, we set each covariate to their overall median value for deep day and shallow night phases.

## Results

### Diel behavior

**Fig 1:**
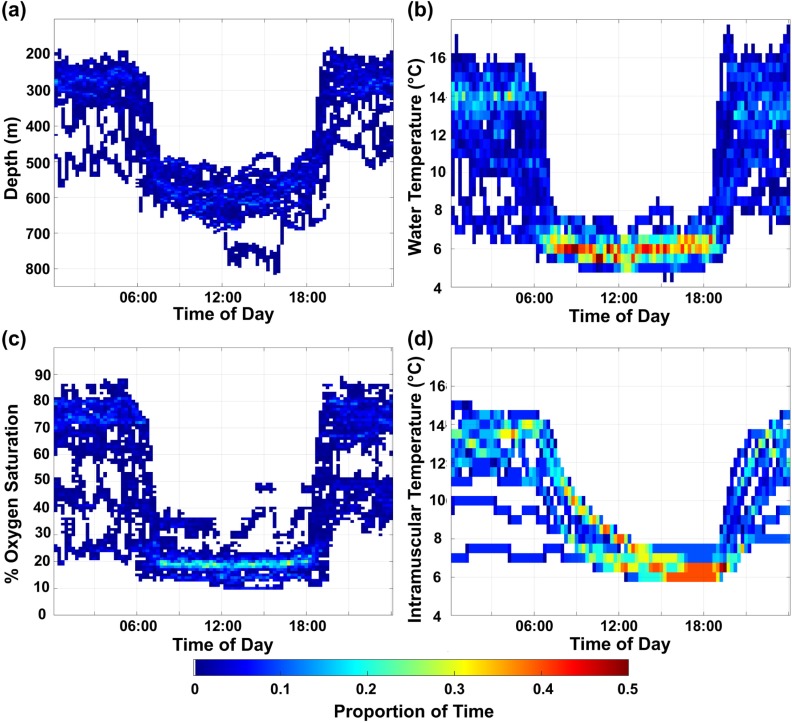
Diel patterns in depth, temperature, and dissolved oxygen. (a) Mean time spent at depth, (b) ambient water temperature, (c) dissolved oxygen saturation, and (d) intramuscular temperature by time of day for all sharks. Plots are color-coded by proportion of time. Proportions greater than 0.5 were observed for (b) only and truncated for clearer illustration.

All sharks undertook diel vertical migrations and spent the majority of their deep daytime distribution (median 11.1 h, IQR 10.5–11.4 h) between 500–650 m (80%) at relatively stable ambient temperatures between 5–7°C (85%) and dissolved oxygen saturations between 10–25% (85%), with a peak between 15–20% (46%) (Fig 1 and S1 Fig). HG5 was the only shark to descend below 700 m and encountered the lowest temperatures (4.7°C) and dissolved oxygen saturations (10%; Table 1); however, only 7% of its overall vertical distribution was below 700 m. During the night, all sharks ascended to shallower waters and spent the majority of their time between 200–350 m (78%), with a peak depth use between 250–300 m (43%) and regularly encountered more variable ambient temperatures between 10–16°C (79%) and dissolved oxygen saturations between 65–80% (55%) (Fig 1 and S1 Fig).

**Fig 2:**
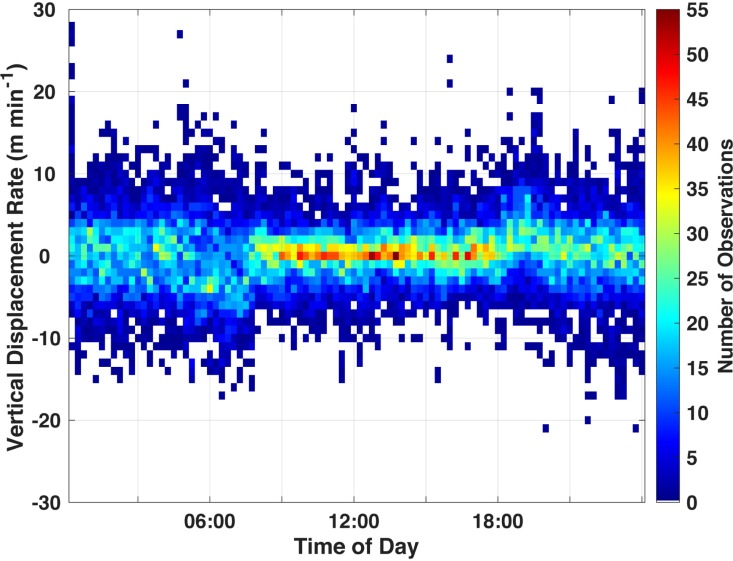
Vertical displacement rate by time of day. Color-coded by number of observations across all sharks. Positive and negative values indicate ascents and descents, respectively.

Sixgill sharks underwent extensive changes in depth (descent: median 297 m, IQR 236–304 m; ascent: median 262 m, IQR 204–284 m) alongside considerable changes in ambient temperature (descent: median 7.1°C, IQR 4.4–7.8°C; ascent: median 5.8°C, IQR 5.1–8.5°C) and dissolved oxygen saturation (descent: median 45%, IQR 32–56%; ascent: median 41%, IQR 31–65%). There was no significant difference (Wilcoxon rank sum test; z = 1.439, *P* = 0.1522) in the duration of dawn descents (median 59 min, IQR 47–80 min) and dusk ascents (median 81 min, IQR 62–100 min). Vertical displacement rates were significantly higher during crepuscular vertical migrations (dawn descents and dusk ascents) compared to shallow night and deep day phases (S1 Table). Overall vertical displacement rates were centered around zero during deep day phases indicating that the sharks were less vertically dynamic compared to shallow night phases where they exhibited a larger range of vertical displacement rates (Fig 2).

### Intramuscular temperature analyses

Sixgill sharks demonstrated a thermal inertia that slowed their rates of heating and cooling across the large changes in ambient water temperature experienced during diel vertical migrations. This thermal inertia is probably attributable to their large body size. Overall, sixgill sharks underwent considerable changes in intramuscular temperature (5.8–14.9°C), but these were slightly narrower than the range of change of ambient water temperatures (4.7–17.7°C). There was no significant difference (Wilcoxon rank sum test; z = 0.3723, *P* = 0.7097) in absolute Δ*T* between the beginning of dawn descents (median 0.4°C, IQR 0.2–1.1°C) and dusk ascents (median 0.4°C, IQR 0.2–0.7°C). Similarly, there was no significant difference (Wilcoxon rank sum test; z = -0.5584, *P* = 0.5765) in absolute Δ*T* between the end of dawn descents (median 4.5°C, IQR 3.5–6.1°C) and dusk ascents (median 5.2°C, 3.3–7.0°C). There was also no significant difference (Wilcoxon rank sum test; z = 0.4964, *P* = 0.6916) in the overall absolute change in intramuscular temperature during dawn descents (median 0.7°C, IQR 0.3–2.1°C) and dusk ascents (median 0.8°C, IQR 0.5–1.2°C).

**Fig 3:**
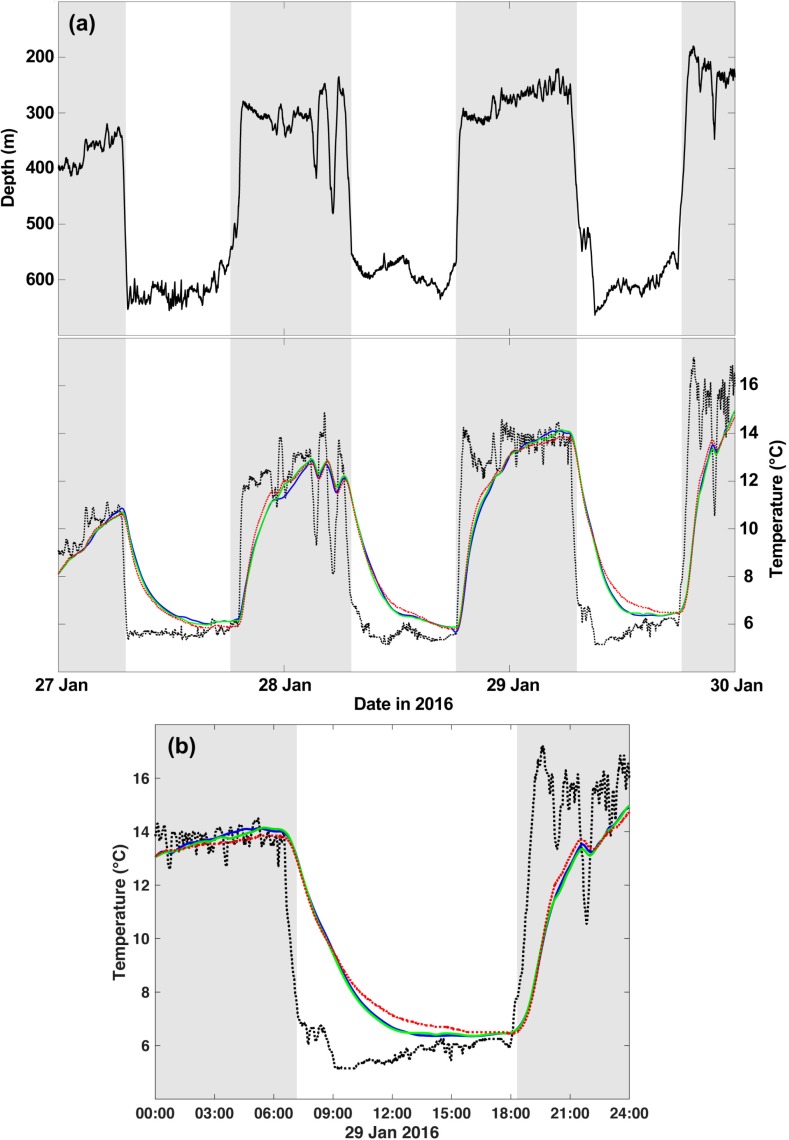
Depth and temperature time series for HG3. Time series of depth (solid black line), ambient water temperature (dotted black line), and intramuscular temperature (colored lines) over (a) 72-h and (b) 24-h periods. Intramuscular temperature colors indicate observed temperatures (dotted red line) and estimated temperatures derived from models using a constant (blue line) and variable (green line) whole-body heat-transfer coefficient. Shaded bars represent night.

**Fig 4:**
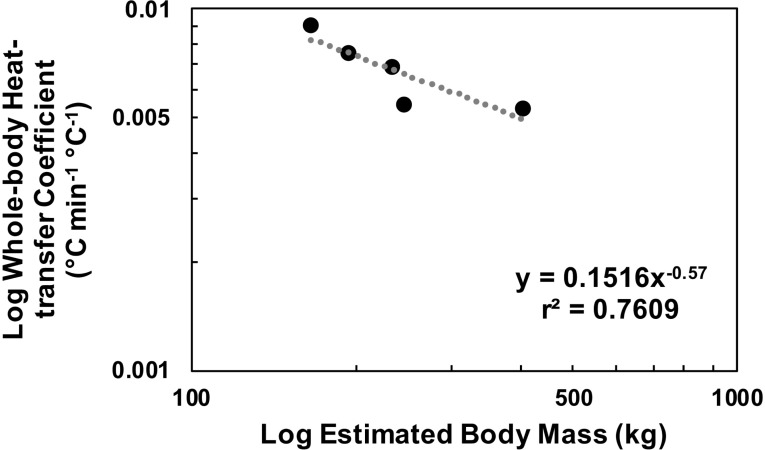
Relationship between estimated body mass of sixgill sharks and optimized constant whole-body heat-transfer coefficients. Dotted gray line represents fitted power regression.

Variable *k* models (2) had lower SSE values compared to constant *k* models (1) for all sharks except HG5 (Table 2). However, intramuscular temperatures estimated by models (1) and (2) were very similar to observed body temperatures (Fig 3) and the confidence intervals for optimized values of *k*_1_ produced by the variable *k* model (2) were relatively large (Table 2), suggesting that these models overfit the data. The output from models (1) and (2) were not significantly different (t-test; *P* > 0.05 for all individuals), and a linear regression of observed intramuscular temperature versus estimated values from constant *k* models (1) explained ≥99% of the variance in intramuscular temperature (slopes 0.99–1.03, y-intercepts -0.21 to 0.10) for all individuals. Therefore, the constant *k* model (1) was selected as the most parsimonious explanation. A comparison of optimized constant *k* values derived from model (1) to estimated body mass indicated that larger individuals had lower whole-body heat-transfer coefficients (Fig 4).

**Table 2:** Results from whole-body heat transfer models. Parameter estimates include ± 95% confidence intervals.

Shark ID	*T_b_* Range (°C)	Model	*k* (°C min^-1^°C^-1^)	*k*_1_ (°C min^-1^°C^-1^)	*k*_2_ (°C min^-1^°C^-1^)	${\dot{T}}_{m}$ (°C min^-1^)	SSE
HG1	6.4–13.9	(1)	0.00546 ± 0.00009			0.00270 ± 0.00003	6025.6
		(2)		0.07264 ± 0.01085	0.00791 ± 0.00006	0.00288 ± 0.00002	5513.0
HG2	6.3–12.1	(1)	0.00531 ± 0.00004			0.00133 ± 0.00001	409.3
		(2)		0.11076 ± 0.04178	0.00694 ± 0.00007	0.00152 ± 0.00002	271.8
HG3	5.8–14.9	(1)	0.00759 ± 0.00006			0.00274 ± 0.00002	1679.7
		(2)		0.014133 ± 0.00054	0.00451 ± 0.00003	0.00133 ± 0.00008	1472.5
HG4	5.9–13.9	(1)	0.00896 ± 0.00008			0.00245 ± 0.00003	844.9
		(2)		0.04497 ± 0.00655	0.00881 ± 0.00007	0.00243 ± 0.00002	696.5
HG5	5.8–14.6	(1)	0.00691 ± 0.00007			0.00189 ± 0.00002	1006.7
		(2)		0.03591 ± 0.01146	0.00541 ± 0.00009	0.00271 ±0.00003	1142.9

*T_b_*, intramuscular temperature; *k*, whole-body heat transfer coefficient; ${\dot{T}}_{m}$, rate of temperature change due to internal (metabolic) heat production; SSE, sum of squared errors.

**Fig 5:**
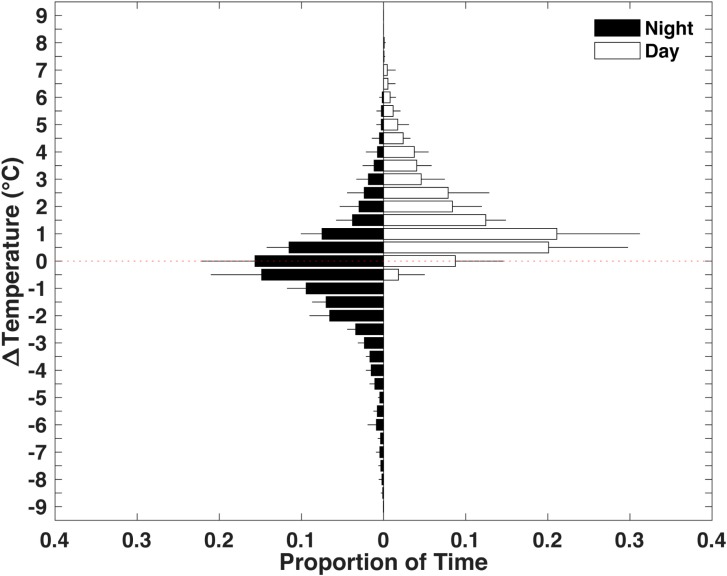
Difference in intramuscular temperature from ambient water temperature (Δ*T*). Mean proportion of time spent at Δ*T* during shallow night and deep day diel phases (excluding dawn descents and dusk ascents) for all individuals. Positive and negative values represent intramuscular temperatures warmer and cooler, respectively, than ambient water temperatures. Error bars represent the standard deviation among individuals.

Sixgill shark intramuscular temperature was >0.5°C above ambient water temperature for the majority (69%) of their deeper daytime distribution (Fig 5). In contrast, intramuscular temperature had a difference of -1.5 to 1.5°C from ambient water temperature for the majority (63%) of their shallower nighttime distribution. HG3 and HG4 experienced the warmest ambient water temperatures of 17.3 and 17.7°C, respectively. However, they spent very little time (2% and 1%) at water temperatures above 16°C. Excursions into warm waters were very brief for HG3 (median 5 min, IQR 3–9 min, maximum 41 min) and HG4 (median 2 min, IQR 1–3 min, maximum 11 min) where median intramuscular temperatures were 3.4°C (IQR 2.2–7.1°C, minimum 1.2°C) and 7.8°C (IQR 5.2–8.6°C, minimum 3.7°C) cooler than ambient water temperatures, respectively.

### Accelerometer data analyses

Acceleration data indicated sixgill sharks (excluding HG4) exhibited relatively shallow pitch angles during dawn descent (-1.5° to -11.8°) and dusk ascent (5.0° to 8.6°) segments of diel vertical migrations (S2 Table). HG1 and HG5 exhibited significantly greater tail beat frequencies during dusk ascents, whereas HG2 and HG3 exhibited higher tail beat frequencies during dawn descents. All individuals had significantly higher amplitude of acceleration (tail beat “effort”) and ODBA during dawn descent than dusk ascents (S2 Table).

Temporal, physiological, and environmental factors were examined for their influence on ODBA. The first principal component (PC1) comprised of depth, ambient water temperature, intramuscular temperature, and dissolved oxygen saturation explained 89.1% of variance (S3 Table) and was included with time of day and swimming phase in the GAMM that best fit the data (S4 Table and S5 Table). The model explained 20.7% of deviance in ODBA by sixgill sharks indicating there are additional factors beyond our model explaining the majority of variation in their activity (S4 Table). Residual analyses indicated a satisfactory fit for small to moderately large ODBA values; however, a departure from normality was observed at very large ODBA values (i.e., bursts) and therefore must be interpreted with caution (S2 Fig).

**Fig 6:**
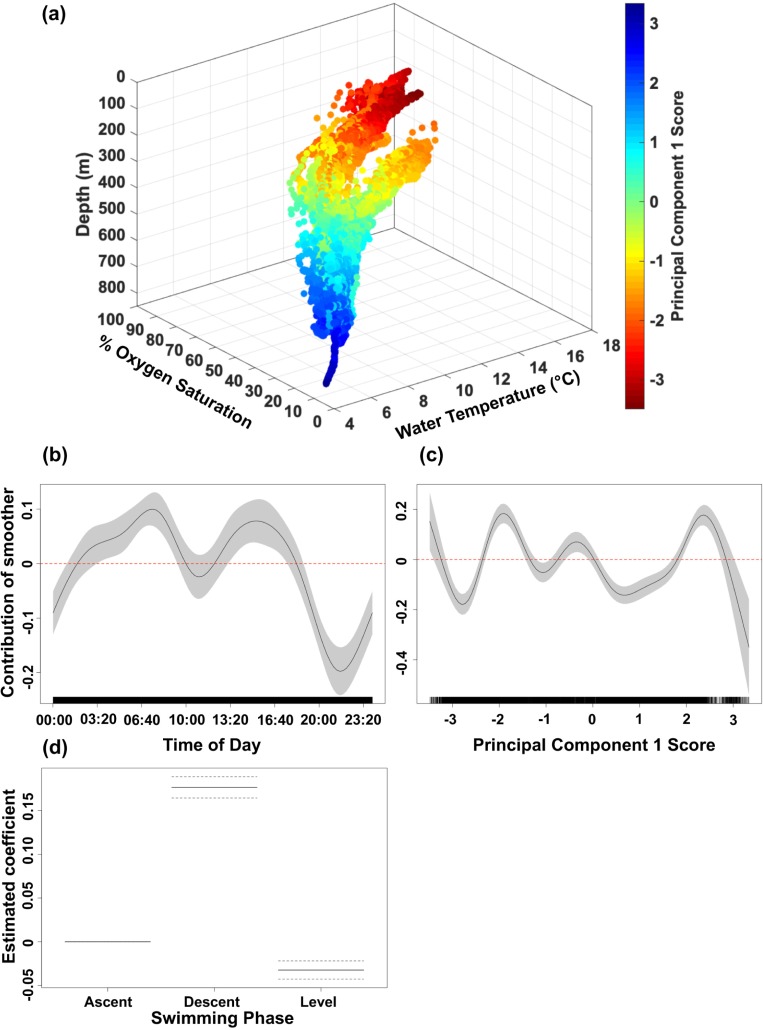
Principal component 1 scores and response curves from the best-fit generalized additive mixed model. (a) Principal component 1 scores (values given by color scale) as distributed in coordinates of co-located ambient water temperature, dissolved oxygen saturation, and depth. (b-c) Estimated response curves (black solid line) of component smooth functions on overall dynamic body acceleration (ODBA) from the best-fit generalized additive mixed model. Shaded areas represent 95% confidence limits of uncertainty in the centered smooth. Vertical axes are partial responses (estimated, centered smooth functions) on the scale of the linear predictor. Ticks on x-axis denote values for which there are data. Positive values on y-axis (above red dashed line) indicate increased ODBA by sixgill sharks. (d) Estimated partial coefficients for the parametric term swimming phase on ODBA from the best-fit model. Base level of ascent is centered, and dashed lines represent 95% confidence limits.

GAMMs revealed sixgill sharks exhibit higher rates of activity during descent compared to ascent and level swimming phases. GAMMs further identified distinct diel patterns in activity by sixgill sharks with an increase in activity from 02:00–10:00 (peak ~07:00) and 12:30–18:00 (peak ~15:00) and reduced activity levels from 18:00–01:30 (low ~21:30) (Fig 6). A peak in activity at PC1 scores ranging from -2.3 to -1.3 corresponded to the shallower nighttime distribution at a median depth of 294 m (IQR 249–277 m), median water temperature of 12.6°C (IQR 12.0–13.3°C), median intramuscular temperature of 11.8°C (IQR 10.7–12.3°C), and median dissolved oxygen saturation of 69% (IQR 67–72%) (Fig 6). An equivalent peak in activity at PC1 scores ranging from 1.8 to 2.8 corresponded to the deeper daytime distribution at a median depth of 604 m (IQR 579–622 m), median water temperature of 5.9°C (IQR 5.6–6.2°C), median intramuscular temperature of 6.8°C (IQR 6.4–7.3°C), and median dissolved oxygen saturation of 18% (IQR 17–20%). ODBA had the greatest decline at PC1 scores >2.8 corresponding to a median depth of 759 m (IQR 747–774 m); however, this result is biased by one individual (HG5) that exceeded a depth of 700 m.

For GAMMs that included swimming phase and time of day with each of the correlated predictor variables, the models including swimming phase, time of day, and depth or water temperature best fit the data (ΔAIC_c_ = 0.8) and explained 15.4% and 17.9% of deviance in ODBA, respectively (S4 Table and S5 Table). Swimming phase and time of day had similar effects on ODBA as the model including PC1 (see above). There was a peak in activity at depths between 550–700 m with a secondary peak between 275–375 m, and activity was lowest at depths shallower than 275 m and below 700 m (HG5 only) (S3 Fig). The highest peak in activity was observed between temperatures of 11–13°C with a secondary peak between 5.0–6.3°C, and a rapid decline at water temperatures exceeding 16°C (S4 Fig).

HMMs were constructed by means of two behavioral states: relatively low levels of ODBA (state 1) and overall higher activity levels including ‘bursts’ in ODBA (state 2) (S5 Fig). The HMM that included individual shark, swimming phase, time of day, dissolved oxygen saturation and the interaction term (time of day × dissolved oxygen saturation) best fit the data (S6 Table). Similar to GAMM residual patterns, HMM pseudo-residual analyses indicated a satisfactory fit for small to moderately large ODBA values and a departure from normality at very large ODBA values (bursts) (S6 Fig). Despite the described lack of fit, the HMM captures most of the relevant structure and these bursts will still be assigned to the high-activity state (state 2) allowing for biologically meaningful inference [48].

**Fig 7:**
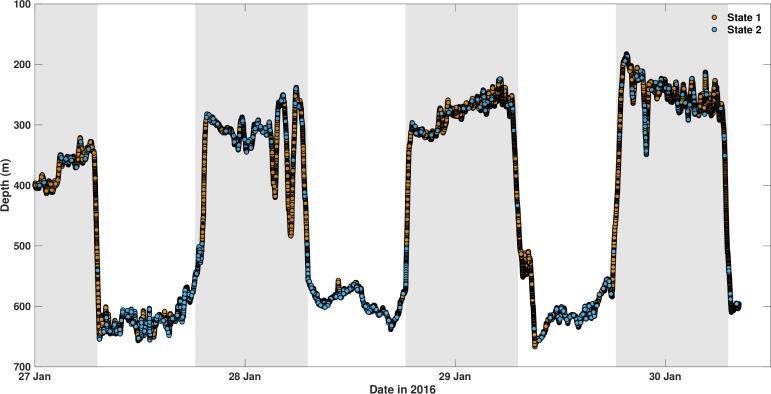
Decoded time series from the best-fit hidden Markov model for HG3. Depth time series with each observation connected to one of the two decoded states. State 1 and state 2 correspond to relatively low and high levels of activity, respectively. Shaded bars represent night.

**Fig 8:**
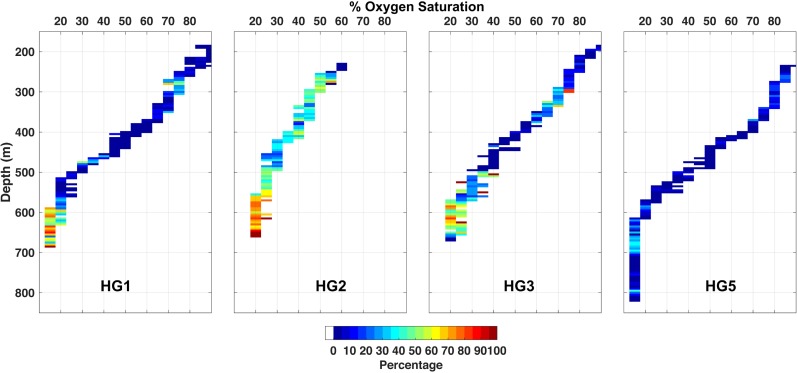
Activity of sixgill sharks along a vertical profile of depth and dissolved oxygen saturation. Each grid cell (0.5 m × 5% saturation) is color-coded by the percentage of observations corresponding to the high-activity state (state 2) from the decoded state sequence derived from the best-fit hidden Markov model.

Overall sixgill sharks spent more time in the less active state (range 66–91%) across their vertical range except for HG2 (47%). Sixgill sharks also remained in the less active state for longer durations (means 8.8–26.3 min, range 1.2–199.3 min) compared to the more active state (means 2.5–9.8 min, range 0.3–180.0 min). Similar to the GAMMs, HMMs identified clear diel patterns in activity with the majority of time spent in the more active state (state 2) during shallow night phases (range 27–60%) and deep day phases (range 29–66%) (Fig 7); however, differences among individuals were observed. Moreover, sixgill sharks spent higher proportions of their deeper daytime distribution (range 36–73%) in the more active state compared to shallower nighttime distributions (range 11–37%) except for HG5 (deep: 6%, shallow: 11%), which spent the least amount of time overall (9%) in the more active state (Fig 8 and S7 Fig).

We estimated the stationary (equilibrium) state probabilities as a function of individual shark, swimming phase, time of day, and dissolved oxygen saturation and set each covariate to their overall median value for deep day (time of day, 12:44:30; dissolved oxygen saturation, 19%) and shallow night phases (time of day, 01:03:30; dissolved oxygen saturation, 71%). Individual shark and swimming phase were set to HG1 and level, respectively. Overall sixgill sharks had a lower probability of being in the high-activity state during ascents when compared to descent and level swimming phases, and during shallow night phases compared to deep day phases. Sixgill sharks showed the highest probabilities of being in the high-activity state between 14:00–18:00 (up to 60% probability) and at low dissolved oxygen saturations below 19% (up to 65% probability) during deep day phases (S8 Fig). The probability of being in the high-activity state is approximately 30% at higher dissolved oxygen saturations (>45%) experienced during shallow night phases and increases in the hours following dusk ascent before declining again to a probability of ~20% prior to dawn descent (S8 Fig).

## Discussion

We combined a novel suite of biologging technologies to examine the fine-scale swimming behavior, activity, and thermal physiology of a relatively understudied deepwater species in relation to its environment. Sixgill sharks undertook pronounced diel vertical migrations and spent a considerable amount of time in cold, low oxygen conditions during their deeper daytime distribution. Analyses of intramuscular temperature revealed sixgill sharks maintain constant heat-transfer coefficients during diel vertical migrations, but their large body mass provides sufficient thermal inertia to slow heat loss during their descent into cold, deep waters. Sixgill sharks exhibited relatively high rates of activity during both shallow night and deep day phases and, contrary to our predictions, did not reduce activity levels during their deeper daytime distribution while they were experiencing low temperature and dissolved oxygen. Further, the highest overall rates of activity were observed during dawn descents and deep day phases.

Biologging technologies overcome the challenges associated with direct observation of behavior, especially for large deep-sea species that are not amenable to captivity [55] and/or suitable for laboratory studies. In particular, high-resolution accelerometers provide a useful tool for examining behavioral responses of free-ranging animals to large changes in environmental conditions. Previous studies examined changes in activity for sixgill sharks using either vertical or horizontal displacement rates via acoustic [56–58] or satellite telemetry [10,59,60]. However, direct comparisons of vertical displacement rates (derived from one-dimensional depth data) and activity (derived from multidimensional acceleration data) have revealed distinct differences in diel patterns [61–63]. For example, [10] demonstrated reduced vertical displacement rates during the deeper daytime distribution of sixgill sharks in Hawai‘i and suggested more active behavior in the shallow nighttime habitat. Here, we demonstrate that although sixgill sharks do exhibit peaks in activity and are more vertically dynamic during their shallower nighttime distribution, they are most active during their deeper daytime distribution. We suggest the decrease in vertical displacement rates during the day may be due to selection of a preferred isobath or to forage on a prey field that is less mobile and dispersed through the water column than prey hunted during the night.

ODBA and amplitude of acceleration were greater during the dawn descent than the dusk ascent segments of diel vertical migrations for all sharks. By contrast, tail beat frequency showed an opposite trend. Although two sharks (HG1 and HG5) displayed higher tail beat frequencies during dusk ascents, consistently higher amplitude of acceleration suggests sharks were beating their tail harder [64] during descents and the higher ODBA values indicate more energy was utilized during descent segments, despite lower tail beat frequencies for two individuals. Although we did not measure and record swimming speed, our findings are consistent with the interpretation that sixgill sharks exert more energy on descent than ascent segments of vertical migrations and periodically glided ‘uphill’ indicative of positive buoyancy [11] (S9 Fig). In addition, sixgill sharks exhibited shallow descent (-1.5° to -11.8°) and ascent angles (5.0° to 8.6°) during vertical migrations typical of movement designed to minimize the cost of horizontal transport [51,65,66]. Intramuscular temperature was close to equilibrium with ambient water temperature at the time of dusk ascent and this cold temperature may necessitate reduced energy expenditure during ascents aided by intermittent passive gliding.

GAMM and HMM analyses revealed diel changes in activity with sixgills most active during their shallow nighttime and deeper daytime distributions. Most notably, GAMMs revealed that sixgill sharks were least active in the warmest waters (>16°C) encountered at shallower depths or in deep waters (>700 m) exposed to low temperature (<5°C) and oxygen conditions (10% saturation). These results are biased by only two individuals (HG3 and HG4) encountering temperatures above 16°C and one individual (HG5) descending below 700 m; however, the lack of occurrence of other individuals at these environmental ranges suggests they may be outside the preferred vertical habitat range for sixgill sharks in Hawai‘i. Moreover, the diel behavior of sixgill sharks in Hawai‘i is fairly predictable and high-resolution (3–15 s) archival records obtained from recovered pop-up satellite tags revealed similar habitat preferences over longer time periods (53–200 days; [10], D. M. Coffey unpublished data). Therefore, the behaviors we observed over shorter time periods are likely representative of long-term behavior. Although sixgill sharks in Hawai‘i have been observed at water temperatures up to 20.6°C, [10] suggested a thermal ceiling at temperatures above 16°C. In other locations, the warmest temperature at which sixgill sharks were observed was 16°C [56] and ~17°C [67]; however, the length of exposure to these temperatures was not reported. Our empirical results support the hypothesis of a preferred thermal habitat at water temperatures below 16°C. This is supported by the fact that the largest decline in activity occurs at temperatures above 16°C and observing that exposures into warmer waters were too short to considerably warm intramuscular temperatures. However, longer duration deployments are needed.

Interestingly, the highest probability of being active was observed during the deeper daytime distribution (500–650 m) at cold temperatures and low oxygen conditions. Sixgill sharks spent the majority of their deeper daytime distribution with intramuscular temperatures warmer than ambient water temperatures providing them with a significant thermal advantage over non-vertically migrating and smaller-sized prey. Therefore, thermal inertia may allow sixgill sharks to maintain higher activity levels for long periods at cold temperatures, and thus exploit deep food resources more effectively compared to more resident species that are at thermal equilibrium. Moreover, oxygen conditions below the thermocline in Hawai‘i are not very variable [10,68] and reach a mean (± SD) saturation of 12 ± 2% (minimum ~9%) between 600–800 m (Station KAHE, Hawai‘i Ocean Time-series [69]) corresponding to the lowest saturation values recorded in this study and [12]. The increase in activity at these depths demonstrates that sixgill sharks are highly tolerant of low oxygen levels occurring within the local OMZ. Though, this activity may incur an oxygen deficit requiring a return to shallower waters with higher oxygen content as suggested for diel vertically-migrating swordfish (*Xiphius gladius*) [6].

Although sixgill sharks exhibited relatively high rates of activity under cold, hypoxic conditions, their large body size and comparatively slow tail beat frequency (mean 0.12–0.21 Hz) and swimming speeds (mean 0.3–0.4 m s^-1^ [11]) suggest they have low metabolic rates relative to more active, shallower-dwelling species [70,71]. Low metabolic rates could allow sixgill sharks to maintain aerobic metabolism under low oxygen conditions [17,18]. However, regional comparisons have revealed that some deep-sea species have low metabolic rates in regions with high oxygen concentrations and exhibit a reduced tolerance for low oxygen conditions (i.e., high critical oxygen thresholds, P_crit_) [17,18]. Therefore, low metabolic rates are not necessarily an explicit adaptation to low oxygen conditions and may better reflect ‘sluggish’ behaviors that result from relaxed selection pressure for rapid locomotory capacities for visual predator-prey interactions in environments with limited visibility [72–75].

Given the greater energy yield of aerobic metabolism, we expect anaerobic metabolism (e.g., anaerobic glycolysis) to merely support activity levels above routine metabolic rates for short periods of time, as opposed to completely supporting metabolic expenditure throughout the daytime distribution under hypoxic conditions [17]. However, the large body size of sixgill sharks and consequent lower mass-specific metabolic rate may allow for greater glycogen stores and a slower accumulation of deleterious anaerobic end-products (e.g., lactate and hydrogen ions [H^+^]) compared to smaller species [76–78]. In addition, sixgill sharks may possess mechanisms for enhanced oxygen extraction and transport, which could enable them to maintain aerobic metabolic rates at the observed low oxygen conditions (i.e., low critical oxygen threshold, P_crit_) [17,18]. For example, some vertical migrators possess high-affinity respiratory proteins (e.g., hemoglobin) that are both temperature and pH sensitive (i.e., Bohr effect), which facilitate oxygen extraction in cold, low oxygenated waters and enhance oxygen release in warm, high oxygen conditions at shallower depths [17,18]. In addition, some residents of OMZs possess adaptations to increase gill diffusion capacities in order to facilitate oxygen uptake in low oxygen conditions including large gill surface areas and thin blood-to-water diffusion distances [17,18,72,79]. Identifying the oxygen partial pressures (pO_2_) at which blood is 50% saturated (P_50_) (e.g., [80]) and the pO_2_ at which sixgill sharks can no longer maintain aerobic metabolism at a level independent of ambient pO_2_ (*P*_crit_) [18] will better define the oxygen tolerance of sixgill sharks, though the latter is technically challenging for large-bodied species, particularly in the deep-sea.

The enhanced thermal inertia of sixgill sharks may confer ecological and physiological advantages at cold temperatures as suggested for fishes with endothermic capacities. Endothermic fishes have evolved specialized anatomical and physiological mechanisms for retaining metabolic heat enabling higher levels of activity for longer durations when diving below the mixed layer to exploit deep forage resources more effectively than ectothermic species [81–83]. However, some large ectothermic sharks also undertake diel vertical migrations into cold temperatures below the thermocline to exploit deep forage resources (e.g., [4,31,84–86]). Though, ectothermic blue sharks (*Prionace glauca*) and ocean sunfish (*Mola mola*) make frequent ascents to surface waters well before intramuscular temperatures reach equilibrium with ambient water temperatures experienced at depth and employ variable heat-transfer coefficients where intramuscular temperatures warm more quickly than it cools [9,84], possibly due to changes in blood flow rates as demonstrated by diel vertically migrating swordfish [87] and bigeye tuna (*Thunnus obesus*) [7,32]. In contrast, sixgill sharks demonstrate prolonged exposures to cold temperatures and low dissolved oxygen concentrations at depth and maintain constant heat-transfer coefficients with comparable rates of heating and cooling, which may be attributed to low metabolic heat production as a result of relatively low activity rates [88,89]. We suggest the combination of relatively slow swimming speeds [11], low activity (as a proxy for low metabolic rates), and large body size with enhanced thermal inertia may enable sixgill sharks to actively search for sparse or patchily distributed forage resources across distinct habitats during diel vertical migrations.

ODBA had many extreme values (i.e., bursts) that needed to be accommodated in both modeling approaches. This issue could be resolved in HMM analyses by estimating the state-dependent densities nonparametrically, particularly for the high-activity state [48,50,90]. Furthermore, the HMMs provide a data-driven, unsupervised learning approach to analyzing ODBA; therefore, we cannot directly link the two activity states to specific behaviors. For example, changes in activity may be the result of foraging, predator avoidance, and/or social interactions, and these behaviors may also not be exclusive to a single state. Moreover, GAMM analyses revealed the best fit model including swimming phase, time of day, and PC1 (comprised of depth, ambient water temperature, intramuscular temperature, and dissolved oxygen saturation) only accounted for 20.7% of deviance in ODBA. Therefore, a significant portion of sixgill shark activity could be explained by an unobserved ecological or physiological covariate such as sporadic feeding events that may be independent of the environmental variables measured here. The relative high rates of activity during both shallow night and deep day diel phases suggests sixgill sharks do not conform to a ‘hunt warm, rest cold’ or ‘hunt cold, rest warm’ behavioral routine [23,24,28]. Disentangling the movement and behavior of sixgill sharks, and other marine species, from their prey remains a significant challenge, especially with limited local dietary information (e.g., see [91]). Recent advances in biologging technologies capable of measuring ingestion and digestion [92] and integrating animal-borne cameras [11,93] would provide meaningful insight into other potential drivers of activity (e.g., foraging) and the adaptive significance of vertical migrations, as well as significantly improve interpretation of these modeling approaches [24,50].

## Conclusions

The novel combination of biologging technologies used in this study provides significant insight into the behavioral and physiological ecology of a deepwater species. The different modeling approaches provide a robust framework for empirically testing the influence of environmental drivers on the activity of sixgill sharks across diel vertical migrations and empirically demonstrated their tolerance of cold, low oxygen conditions in deepwater habitats. Obtaining similar data in regions with more variable and/or lower dissolved oxygen content may help define the oxygen tolerance for this species and resolve the relative impacts of cold temperatures and low oxygen on behavior [17,72]. Significant advances in biologging technologies enable innovative *in situ* deep-sea natural experiments, thus improving our understanding of the biological response of deepwater species to contemporary and future ocean conditions.

## Supporting information

S1 Fig Diel patterns in habitat use. (PDF)

S2 Fig Diagnostic plots for the best-fit generalized additive mixed model on overall dynamic body acceleration. (PDF)

S3 Fig Response curves and partial coefficients from the generalized additive mixed model including swimming phase, time of day, and depth. (PDF)

S4 Fig Response curves and partial coefficients from the generalized additive mixed model including swimming phase, time of day, and water temperature. (PDF)

S5 Fig State-dependent densities of overall dynamic body acceleration (ODBA) for sixgill sharks. (PDF)

S6 Fig Diagnostic plots for the two-state hidden Markov model with the best fit on overall dynamic body acceleration. (PDF)

S7 Fig Activity of sixgill sharks along a vertical profile of depth and ambient water temperature. (PDF)

S8 Fig Stationary state probabilities from the best-fit hidden Markov model. (PDF)

S9 Fig Swimming performance during a vertical movement. (PDF)

S1 Table Results of Kruskal-Wallis test examining the influence of diel depth phase on vertical displacement rate. (PDF)

S2 Table Results of Kruskal-Wallis test examining the influence of diel depth phase on pitch and acceleration metrics. (PDF)

S3 Table Principal component analysis (PCA) loadings for components (1‐4). (PDF)

S4 Table Ranked generalized additive mixed models of effects on overall dynamic body acceleration. (PDF)

S5 Table Results from the best generalized additive mixed models fitted to overall dynamic body acceleration (ODBA). (PDF)

S6 Table Ranked hidden Markov models of effects on activity states based on overall dynamic body acceleration. (PDF)

## References

[1] Neilson JD, Perry RI. Diel vertical migrations of marine fishes: an obligate or facultative process? Advances in Marine Biology. 1990;26:115–68. 10.1016/s0065-2881(08)60200-x

[2] Hays GC. A review of the adaptive significance and ecosystem consequences of zooplankton diel vertical migrations. Hydrobiologia. 2003;503:163–70.

[3] Scheuerell MD, Schindler DE. Diel vertical migration by juvenile sockeye salmon: empirical evidence for the antipredation window. Ecology. 2003;84(7):1713–20.

[4] Sims DW, Southall EJ, Tarling GA, Metcalfe JD. Habitat-specific normal and reverse diel vertical migration in the plankton-feeding basking shark. Journal of Animal Ecology. 2005;74(4):755–61.

[5] Mehner T. Diel vertical migration of freshwater fishes—proximate triggers, ultimate causes and research perspectives. Freshwater Biology. 2012;57(7):1342–59.

[6] Carey FG, Robison BH. Daily patterns in the activities of swordfish, *Xiphias gladius*, observed by acoustic telemetry. Fishery Bulletin. 1981;79(2):277–92.

[7] Holland KN, Brill RW, Chang RKC, Sibert JR, Fournier DA. Physiological and behavioural thermoregulation in bigeye tuna (*Thunnus obesus*). Nature. 1992;358:410–2. 10.1038/358410a0 1641023

[8] Gilly WF, Zeidberg LD, Booth JA, Stewart JS, Marshall G, Abernathy K, et al Locomotion and behavior of Humboldt squid, *Dosidicus gigas*, in relation to natural hypoxia in the Gulf of California, Mexico. The Journal of experimental biology. 2012;215(18):3175–90. 22915711 10.1242/jeb.072538

[9] Nakamura I, Goto Y, Sato K. Ocean sunfish rewarm at the surface after deep excursions to forage for siphonophores. Journal of Animal Ecology. 2015;84(3):590–603. 10.1111/1365-2656.12346 25643743

[10] Comfort CM, Weng KC. Vertical habitat and behaviour of the bluntnose sixgill shark in Hawaii. Deep Sea Research Part II: Topical Studies in Oceanography. 2015;115:116–26.

[11] Nakamura I, Meyer CG, Sato K. Unexpected positive buoyancy in deep sea sharks, *Hexanchus griseus*, and a *Echinorhinus cookei*. PloS one. 2015;10(6):e0127667 10.1371/journal.pone.0127667 26061525 PMC4489517

[12] Coffey DM, Holland KN. First autonomous recording of in situ dissolved oxygen from free-ranging fish. Animal Biotelemetry. 2015;3:47.

[13] Vaquer-Sunyer R, Duarte CM. Thresholds of hypoxia for marine biodiversity. Proceedings of the National Academy of Sciences. 2008;105(40):15452–7. 10.1073/pnas.0803833105 PMC2556360 18824689

[14] Ekau W, Auel H, Pörtner HO, Gilbert D. Impacts of hypoxia on the structure and processes in pelagic communities (zooplankton, macro-invertebrates and fish). Biogeosciences. 2010;7(5):1669–99.

[15] Hofmann AF, Peltzer ET, Walz PM, Brewer PG. Hypoxia by degrees: establishing definitions for a changing ocean. Deep Sea Research Part I: Oceanographic Research Papers. 2011;58(12):1212–26.

[16] Yeh J, Drazen JC. Depth zonation and bathymetric trends of deep-sea megafaunal scavengers of the Hawaiian Islands. Deep Sea Research Part I: Oceanographic Research Papers. 2009;56(2):251–66.

[17] Childress JJ, Seibel BA. Life at stable low oxygen levels: adaptations of animals to oceanic oxygen minmum layers. Journal of Experimental Biology. 1998;201(8):1223–32. 9510533 10.1242/jeb.201.8.1223

[18] Seibel BA. Critical oxygen levels and metabolic suppression in oceanic oxygen minimum zones. Journal of Experimental Biology. 2011;214(2):326–36. 21177952 10.1242/jeb.049171

[19] Huey RB, Kingsolver JG. Evolution of thermal sensitivity of ectotherm performance. Trends in Ecology & Evolution. 1989;4(5):131–5. 21227334 10.1016/0169-5347(89)90211-5

[20] Dell AI, Pawar S, Savage VM. Systematic variation in the temperature dependence of physiological and ecological traits. Proceedings of the National Academy of Sciences. 2011;108(26):10591–6. 10.1073/pnas.1015178108 PMC3127911 21606358

[21] Payne NL, Smith JA, van der Meulen DE, Taylor MD, Watanabe YY, Takahashi A, et al Temperature dependence of fish performance in the wild: links with species biogeography and physiological thermal tolerance. Functional Ecology. 2016;30(6):903–12.

[22] Dell AI, Pawar S, Savage VM. Temperature dependence of trophic interactions are driven by asymmetry of species responses and foraging strategy. Journal of Animal Ecology. 2014;83(1):70–84. 10.1111/1365-2656.12081 23692182

[23] Sims DW, Wearmouth VJ, Southall EJ, Hill JM, Moore P, Rawlinson K, et al Hunt warm, rest cool: bioenergetic strategy underlying diel vertical migration of a benthic shark. Journal of Animal Ecology. 2006;75(1):176–90. 10.1111/j.1365-2656.2005.01033.x 16903055

[24] Papastamatiou YP, Watanabe YY, Bradley D, Dee LE, Weng K, Lowe CG, et al Drivers of daily routines in an ectothermic marine predator: hunt warm, rest warmer? PloS one. 2015;10(6):e0127807 10.1371/journal.pone.0127807 26061229 PMC4489509

[25] Neill WH, Stevens ED, Carey FG, Lawson KD, Mrosovsky N, Frair W. Thermal inertia versus thermoregulation in "warm" turtles and tunas. Science. 1974;184(4140):1008–10. 10.1126/science.184.4140.1008 4826167

[26] Neill WH, Chang RKC, Dizon AE. Magnitude and ecological implications of thermal inertia in skipjack tuna, *Katsuwonus pelamis* (Linnaeus). Environmental Biology of Fishes. 1976;1(1):61–80.

[27] Wurtsbaugh WA, Neverman D. Post-feeding thermotaxis and daily vertical migration in a larval fish. Nature. 1988;333(6176):846–8.

[28] Di Santo V, Bennett WA. Is post-feeding thermotaxis advantageous in elasmobranch fishes? Journal of Fish Biology. 2011;78(1):195–207. 10.1111/j.1095-8649.2010.02853.x 21235555

[29] Ebert DA. Some observations on the reproductive biology of the sixgill shark *Hexanchus griseus* (Bonnaterre, 1788) from southern African waters. African Journal of Marine Science. 2002;24:359–63.

[30] Ebert DA, editor Aspects on the biology of hexanchoid sharks along the California coast. Second International Conference on Indo-Pacific Fishes; 1986; Tokyo: Ichthyological Society of Japan.

[31] Nelson DR, McKibben JN, Strong WR, Lowe CG, Sisneros JA, Schroeder DM, et al An acoustic tracking of a megamouth shark, *Megachasma pelagios*: a crepuscular vertical migrator. Environmental Biology of Fishes. 1997;49(4):389–99.

[32] Holland KN, Sibert JR. Physiological thermoregulation in bigeye tuna. Environmental Biology of Fishes. 1994;40(3):319–27.

[33] Sakamoto KQ, Sato K, Ishizuka M, Watanuki Y, Takahashi A, Daunt F, et al Can ethograms be automatically generated using body acceleration data from free-ranging birds? PloS one. 2009;4(4):e5379 10.1371/journal.pone.0005379 19404389 PMC2671159

[34] Collins PM, Green JA, Warwick-Evans V, Dodd S, Shaw PJ, Arnould JP, et al Interpreting behaviors from accelerometry: a method combining simplicity and objectivity. Ecology and Evolution. 2015;5(20):4642–54. 10.1002/ece3.1660 26668729 PMC4670056

[35] Kawatsu S, Sato K, Watanabe Y, Hyodo S, Breves JP, Fox BK, et al A New Method to Calibrate Attachment Angles of Data Loggers in Swimming Sharks. EURASIP Journal on Advances in Signal Processing. 2010;2010:732586.

[36] Nakamura I, Watanabe YY, Papastamatiou YP, Sato K, Meyer CG. Yo-yo vertical movements suggest a foraging strategy for tiger sharks *Galeocerdo cuvier*. Marine Ecology Progress Series. 2011;424:237–46.

[37] Wilson RP, White CR, Quintana F, Halsey LG, Liebsch N, Martin GR, et al Moving towards acceleration for estimates of activity-specific metabolic rate in free-living animals: the case of the cormorant. Journal of Animal Ecology. 2006;75(5):1081–90. 10.1111/j.1365-2656.2006.01127.x 16922843

[38] Halsey LG, Shepard EL, Quintana F, Gomez Laich A, Green JA, Wilson RP. The relationship between oxygen consumption and body acceleration in a range of species. Comparative Biochemistry and Physiology Part A: Molecular & Integrative Physiology. 2009;152(2):197–202. 10.1016/j.cbpa.2008.09.021 18854225

[39] Gleiss AC, Wilson RP, Shepard ELC. Making overall dynamic body acceleration work: on the theory of acceleration as a proxy for energy expenditure. Methods in Ecology and Evolution. 2011;2(1):23–33.

[40] Qasem L, Cardew A, Wilson A, Griffiths I, Halsey LG, Shepard EL, et al Tri-axial dynamic acceleration as a proxy for animal energy expenditure; should we be summing values or calculating the vector? PloS one. 2012;7(2):e31187 10.1371/journal.pone.0031187 22363576 PMC3281952

[41] Zuur AF, Ieno EN, Elphick CS. A protocol for data exploration to avoid common statistical problems. Methods in Ecology and Evolution. 2010;1(1):3–14.

[42] Zuur AF, Ieno EN, Walker NJ, Saveliev AA, Smith GM. Mixed Effects Models and Extensions in Ecology with R. Gail M, Krickeberg K, Samet JM, Tsiatis A, Wong W, editors. New York, NY: Springer; 2009 574 p.

[43] R Core Team. R: A language and environment for statistical computing. Vienna, Austria: R Foundation for Statistical Computing; 2018.

[44] Wood SN, Scheipl F, Faraway JJ. Straightforward intermediate rank tensor product smoothing in mixed models. Statistics and Computing. 2013;23(3):341–60.

[45] Wood SN. Generalized Additive Models: An Introduction with R. Second ed Boca Raton, FL: CRC Press; 2017.

[46] Burnham KP, Anderson DR. Model Selection and Multimodel Inference: A Practical Information-Theoretic Approach. 2nd ed New York, NY: Springer; 2002 496 p.

[47] Langrock R, King R, Matthiopoulos J, Thomas L, Fortin D, Morales JM. Flexible and practical modeling of animal telemetry data: hidden Markov models and extensions. Ecology. 2012;93(11):2336–42. 10.1890/11-2241.1 23236905

[48] Leos-Barajas V, Photopoulou T, Langrock R, Patterson TA, Watanabe YY, Murgatroyd M, et al Analysis of animal accelerometer data using hidden Markov models. Methods in Ecology and Evolution. 2017;8(2):161–73.

[49] Patterson TA, Basson M, Bravington MV, Gunn JS. Classifying movement behaviour in relation to environmental conditions using hidden Markov models. Journal of Animal Ecology. 2009;78(6):1113–23. 10.1111/j.1365-2656.2009.01583.x 19563470

[50] Papastamatiou YP, Watanabe YY, Demsar U, Leos-Barajas V, Bradley D, Langrock R, et al Activity seascapes highlight central place foraging strategies in marine predators that never stop swimming. Movement Ecology. 2018;6:9 10.1186/s40462-018-0127-3 29951206 PMC6011523

[51] Gleiss AC, Norman B, Wilson RP. Moved by that sinking feeling: variable diving geometry underlies movement strategies in whale sharks. Functional Ecology. 2011;25(3):595–607.

[52] Gleiss AC, Gruber SH, Wilson RP. Multi-channel data-logging: towards determination of behaviour and metabolic rate in free-swimming sharks. In: Nielsen JL, Arrizabalaga H, Fragoso N, Hobday A, Lutcavage M, Sibert J, editors. Tagging and Tracking of Marine Animals with Electronic Devices. Reviews: Methods and Technologies in Fish Biology and Fisheries. 9 Dordrecht, Netherlands: Springer; 2009 p. 211–28.

[53] McClintock BT, Michelot T. momentuHMM: R package for generalized hidden Markov models of animal movement. Methods in Ecology and Evolution. 2018;9(6):1518–30.

[54] Zucchini W, MacDonald IL, Langrock R. Hidden Markov Models for Time Series: An Introduction Using R. Second ed Boca Raton, FL: CRC Press; 2016.

[55] Grassmann M, McNeil B, Wharton J. Sharks in captivity: the role of husbandry, breeding, education, and citizen science in shark conservation. In: Larson SE, Lowry D, editors. Northeast Pacific Shark Biology, Research and Conservation Part B. Advances in Marine Biology 78: Academic Press; 2017 p. 89–119. 10.1016/bs.amb.2017.08.002 29056144

[56] Carey FG, Clark E. Depth telemetry from the sixgill shark, *Hexanchus griseus*, at Bermuda. Environmental Biology of Fishes. 1995;42(1):7–14.

[57] Andrews KS, Levin PS, Katz SL, Farrer D, Gallucci VF, Bargmann G. Acoustic monitoring of sixgill shark movements in Puget Sound: evidence for localized movement. Canadian Journal of Zoology. 2007;85(11):1136–43.

[58] Andrews KS, Williams GD, Farrer D, Tolimieri N, Harvey CJ, Bargmann G, et al Diel activity patterns of sixgill sharks, *Hexanchus griseus*: the ups and downs of an apex predator. Animal Behaviour. 2009;78(2):525–36.

[59] King JR, Surry AM. Seasonal and daily movements of the bluntnose sixgill shark (*Hexanchus griseus*) in the strait of Georgia from satellite tag data. Environmental Biology of Fishes. 2017;100(12):1543–59.

[60] Rodríguez-Cabello C, González-Pola C, Rodríguez A, Sánchez F. Insights about depth distribution, occurrence and swimming behavior of *Hexanchus griseus* in the Cantabrian Sea (NE Atlantic). Regional Studies in Marine Science. 2018;23:60–72.

[61] Gleiss AC, Wright S, Liebsch N, Wilson RP, Norman B. Contrasting diel patterns in vertical movement and locomotor activity of whale sharks at Ningaloo Reef. Marine Biology. 2013;160(11):2981–92.

[62] Gleiss AC, Morgan DL, Whitty JM, Keleher JJ, Fossette S, Hays GC. Are vertical migrations driven by circadian behaviour? Decoupling of activity and depth use in a large riverine elasmobranch, the freshwater sawfish (*Pristis pristis*). Hydrobiologia. 2016;787(1):181–91.

[63] Whitney NM, Lear KO, Gleiss AC, Payne N, White CF. Advances in the application of high-resolution biologgers to elasmobranch fishes In: Carrier JC, Heithaus MR, Simpfendorfer CA, editors. Shark Research: Emerging Technologies and Applications for the Field and Laboratory. CRC Marine Biology Series. Boca Raton, FL: CRC Press; 2018 p. 45–69.

[64] Whitney NM, White CF, Gleiss AC, Schwieterman GD, Anderson P, Hueter RE, et al A novel method for determining post-release mortality, behavior, and recovery period using acceleration data loggers. Fisheries Research. 2016;183:210–21.

[65] Weihs D. Mechanically efficient swimming techniques for fish with negative buoyancy. Journal of Marine Research. 1973;31:194–209.

[66] Papastamatiou YP, Iosilevskii G, Leos-Barajas V, Brooks EJ, Howey LA, Chapman DD, et al Optimal swimming strategies and behavioral plasticity of oceanic whitetip sharks. Scientific Reports. 2018;8(1):551 10.1038/s41598-017-18608-z 29323131 PMC5765167

[67] Grubbs RD, Cotton C, Daly-Engel T, Kerstetterm D. Post-release survival and vertical movements of bluntnose sixgill sharks (*Hexanchus griseus*) in four oceanic regions. 30th American Elasmobranch Society Symposium; Chattanooga, TN2014.

[68] Bingham FM, Lukas R. Seasonal cycles of temperature, salinity and dissolved oxygen observed in the Hawaii Ocean Time-series. Deep Sea Research Part II: Topical Studies in Oceanography. 1996;43(2):199–213.

[69] Karl DM, Lukas R. The Hawaii Ocean Time-series (HOT) program: background, rationale and field implementation. Deep Sea Research Part II: Topical Studies in Oceanography. 1996;43(2):129–56.

[70] Watanabe YY, Lydersen C, Fisk AT, Kovacs KM. The slowest fish: swim speed and tail-beat frequency of Greenland sharks. Journal of Experimental Marine Biology and Ecology. 2012;426–427:5–11.

[71] Jacoby DM, Siriwat P, Freeman R, Carbone C. Is the scaling of swim speed in sharks driven by metabolism? Biology letters. 2015;11(12):20150781 10.1098/rsbl.2015.0781 26631246 PMC4707698

[72] Childress JJ. Are there physiological and biochemical adaptations of metabolism in deep-sea animals? Trends in Ecology & Evolution. 1995;10(1):30–6. 21236941 10.1016/s0169-5347(00)88957-0

[73] Drazen JC, Seibel BA. Depth-related trends in metabolism of benthic and benthopelagic deep-sea fishes. Limnology and Oceanography. 2007;52(5):2306–16.

[74] Seibel BA, Drazen JC. The rate of metabolism in marine animals: environmental constraints, ecological demands and energetic opportunities. Philosophical Transactions of the Royal Society B: Biological Sciences. 2007;362(1487):2061–78. 10.1098/rstb.2007.2101 17510016 PMC2442854

[75] Condon NE, Friedman JR, Drazen JC. Metabolic enzyme activities in shallow- and deep-water chondrichthyans: implications for metabolic and locomotor capacity. Marine Biology. 2012;159(8):1713–31.

[76] Almeida-Val VM, Val AL, Duncan WP, Souza FC, Paula-Silva MN, Land S. Scaling effects on hypoxia tolerance in the Amazon fish *Astronotus ocellatus* (Perciformes: Cichlidae): contribution of tissue enzyme levels. Comparative Biochemistry and Physiology Part B: Biochemistry and Molecular Biology. 2000;125(2):219–26. 10.1016/s0305-0491(99)00172-8 10817909

[77] Nilsson GE, Östlund‐Nilsson S. Does size matter for hypoxia tolerance in fish? Biological Reviews. 2008;83(2):173–89. 10.1111/j.1469-185X.2008.00038.x 18397180

[78] Urbina MA, Glover CN. Relationship between fish size and metabolic rate in the oxyconforming inanga *Galaxias maculatus* reveals size-dependent strategies to withstand hypoxia. Physiological and Biochemical Zoology. 2013;86(6):740–9. 10.1086/673727 24241070

[79] Friedman JR, Condon NE, Drazen JC. Gill surface area and metabolic enzyme activities of demersal fishes associated with the oxygen minimum zone off California. Limnology and Oceanography. 2012;57(6):1701–10.

[80] Herbert NA, Skov PV, Tirsgaard B, Bushnell PG, Brill RW, Harvey Clark C, et al Blood O2 affinity of a large polar elasmobranch, the Greenland shark *Somniosus microcephalus*. Polar Biology. 2017;40(11):2297–305.

[81] Weng KC, Castilho PC, Morrissette JM, Landeira-Fernandez AM, Holts DB, Schallert RJ, et al Satellite tagging and cardiac physiology reveal niche expansion in salmon sharks. Science. 2005;310(5745):104–6. 10.1126/science.1114616 16210538

[82] Madigan DJ, Carlisle AB, Gardner LD, Jayasundara N, Micheli F, Schaefer KM, et al Assessing niche width of endothermic fish from genes to ecosystem. Proceedings of the National Academy of Sciences. 2015;112(27):8350–5. 10.1073/pnas.1500524112 PMC4500250 26100889

[83] Watanabe YY, Goldman KJ, Caselle JE, Chapman DD, Papastamatiou YP. Comparative analyses of animal-tracking data reveal ecological significance of endothermy in fishes. Proceedings of the National Academy of Sciences. 2015;112(19):6104–9. 10.1073/pnas.1500316112 PMC4434765 25902489

[84] Carey FG, Scharold JV. Movements of blue sharks (*Prionace glauca*) in depth and course. Marine Biology. 1990;106:329–42.

[85] West GJ, Stevens JD. Archival tagging of school shark, *Galeorhinus galeus*, in Australia—initial results. Environmental Biology of Fishes. 2001;60:283–98.

[86] Hulbert LB, Sigler MF, Lunsford CR. Depth and movement behaviour of the Pacific sleeper shark in the north-east Pacific Ocean. Journal of Fish Biology. 2006;69(2):406–25.

[87] Stoehr A, St. Martin J, Aalbers S, Sepulveda C, Bernal D, Eliason EJ. Free-swimming swordfish, *Xiphias gladius*, alter the rate of whole body heat transfer: morphological and physiological specializations for thermoregulation. ICES Journal of Marine Science. 2018;75(2):858–70.

[88] Hight BV, Lowe CG. Elevated body temperatures of adult female leopard sharks, *Triakis semifasciata*, while aggregating in shallow nearshore embayments: evidence for behavioral thermoregulation? Journal of Experimental Marine Biology and Ecology. 2007;352(1):114–28.

[89] Meekan MG, Fuiman LA, Davis R, Berger Y, Thums M. Swimming strategy and body plan of the world's largest fish: implications for foraging efficiency and thermoregulation. Frontiers in Marine Science. 2015;2:64.

[90] Langrock R, Kneib T, Sohn A, DeRuiter SL. Nonparametric inference in hidden Markov models using P-splines. Biometrics. 2015;71(2):520–8. 10.1111/biom.12282 25586063

[91] Crow GL, Lowe CG, Wetherbee BM. Shark records from longline fishing programs in Hawai'i with comments on Pacific Ocean distributions. Pacific Science. 1996;50(4):382–92.

[92] Meyer CG, Holland KN. Autonomous measurement of ingestion and digestion processes in free-swimming sharks. Journal of Experimental Biology. 2012;215(21):3681–4. 22855614 10.1242/jeb.075432

[93] Papastamatiou YP, Meyer CG, Watanabe YY, Heithaus MR. Animal-borne video cameras and their use to study shark ecology and conservation In: Carrier JC, Heithaus MR, Simpfendorfer CA, editors. Shark Research: Emerging Technologies and Applications for the Field and Laboratory. CRC Marine Biology Series. Boca Raton, FL: CRC Press; 2018 p. 83–92.

