# Supplementary material for: Diel patterns in swimming behavior of a vertically migrating deepwater shark, the bluntnose sixgill (Hexanchus griseus)
Source: PLoS One. 2020 Jan 24;15(1):e0228253. doi: 10.1371/journal.pone.0228253 (PMC6980647; doi:10.1371/journal.pone.0228253)
Supplement: S1 Fig — (PDF) [file pone.0228253.s001.pdf]

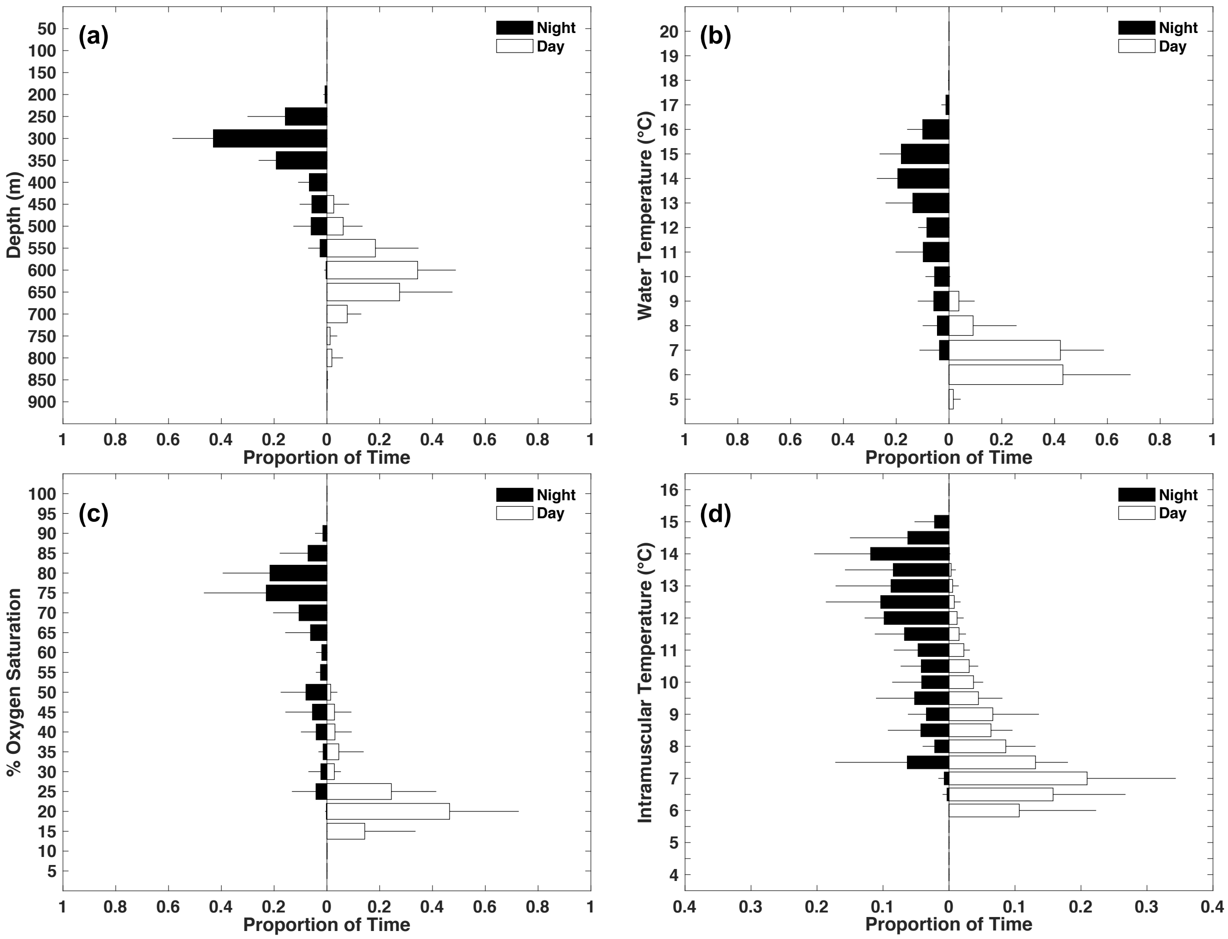

S1 Fig. Diel patterns in habitat use. Mean proportion of time spent at (a) depth, (b) ambient water temperature, (c) dissolved oxygen saturation, and (d) intramuscular temperature during shallow night and deep day diel phases (excluding dawn descents and dusk ascents) for all individuals. Error bars represent the standard deviation among individuals.
