# Supplementary material for: Diel patterns in swimming behavior of a vertically migrating deepwater shark, the bluntnose sixgill (Hexanchus griseus)
Source: PLoS One. 2020 Jan 24;15(1):e0228253. doi: 10.1371/journal.pone.0228253 (PMC6980647; doi:10.1371/journal.pone.0228253)
Supplement: S2 Fig — (PDF) [file pone.0228253.s002.pdf]

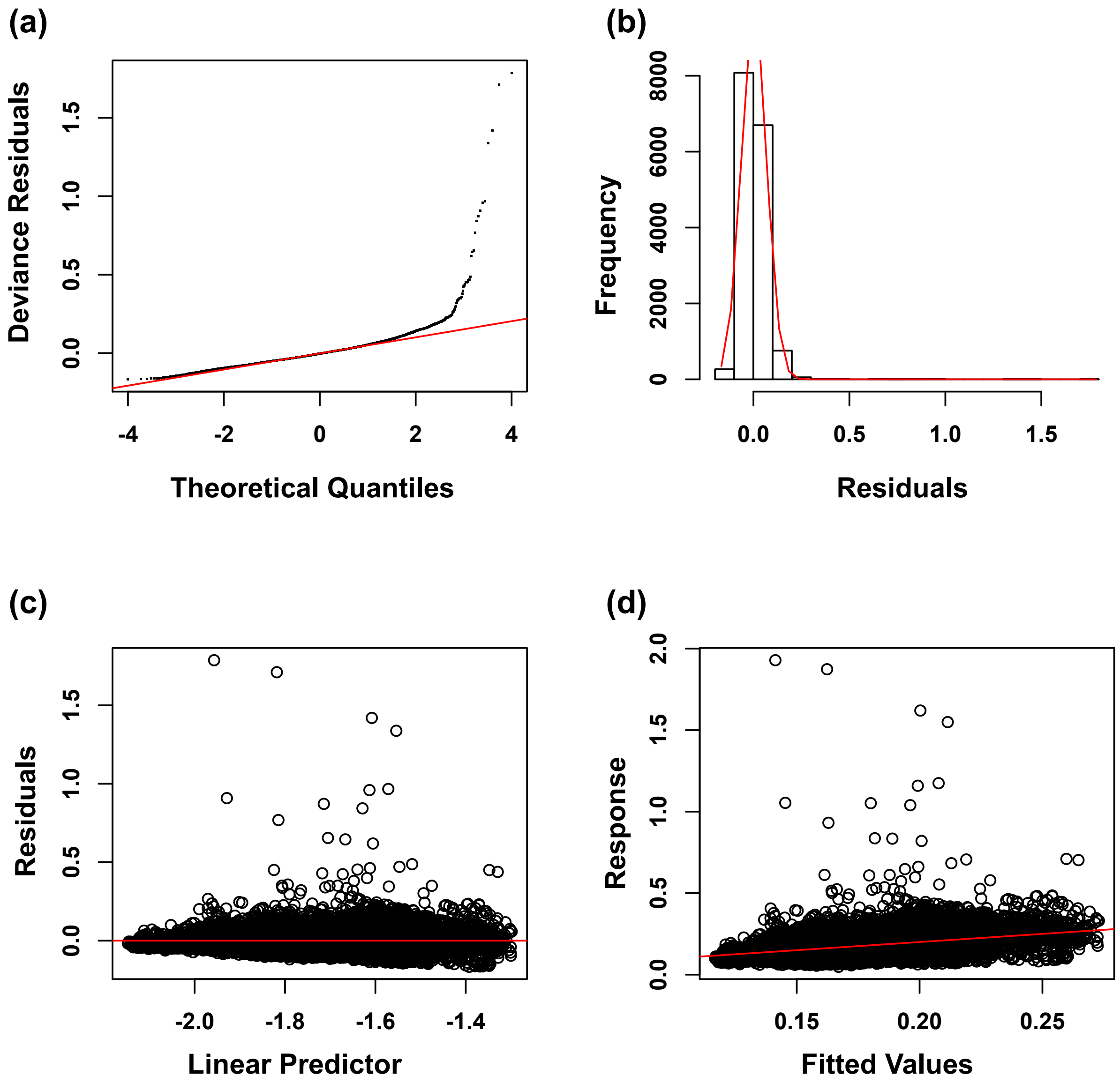


**S2 Fig. Diagnostic plots for the best-fit generalized additive mixed model on overall dynamic body acceleration.** (a) Quantile-quantile (Q-Q) plot for deviance residuals of the model with 1:1 line (red). (b) Distribution of normalized residuals with fitted normal curve (red). (c) Residuals versus linear predictor with zero-centered line (red). (d) Fitted values versus observed values with 1:1 line (red).
