# Supplementary material for: Diel patterns in swimming behavior of a vertically migrating deepwater shark, the bluntnose sixgill (Hexanchus griseus)
Source: PLoS One. 2020 Jan 24;15(1):e0228253. doi: 10.1371/journal.pone.0228253 (PMC6980647; doi:10.1371/journal.pone.0228253)
Supplement: S3 Fig — (PDF) [file pone.0228253.s003.pdf]

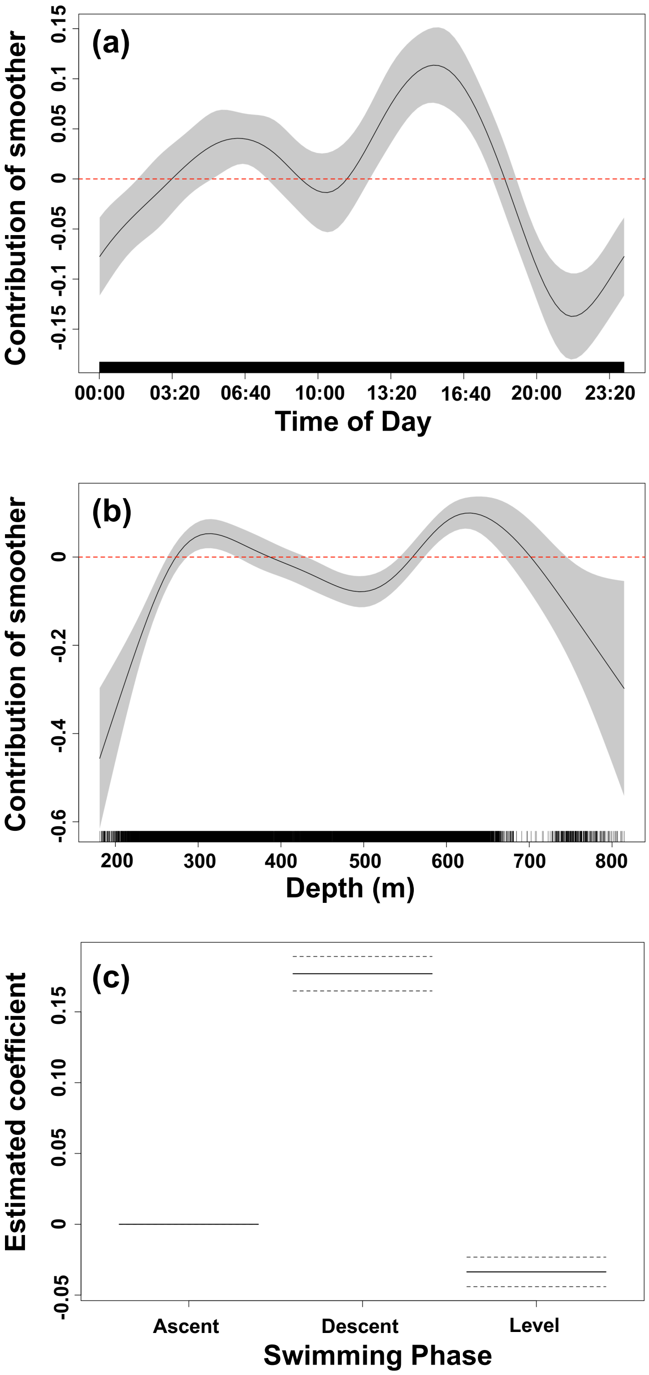

S3 Fig. Response curves and partial coefficients from the generalized additive mixed model including swimming phase, time of day, and depth. (a-b) Estimated response curves (black solid line) of component smooth functions on overall dynamic body acceleration (ODBA). Shaded areas represent 95% confidence limits of uncertainty in the centered smooth. Vertical axes are partial responses (estimated, centered smooth functions) on the scale of the linear predictor. Ticks on x-axis denote values for which there are data. Positive values on y-axis (above red dashed line) indicate increased ODBA by sixgill sharks. (c) Estimated partial coefficients for the parametric term swimming phase on ODBA from the best-fit model. Base level of ascent is centered and dashed lines represent 95% confidence limits.
