# Supplementary material for: Diel patterns in swimming behavior of a vertically migrating deepwater shark, the bluntnose sixgill (Hexanchus griseus)
Source: PLoS One. 2020 Jan 24;15(1):e0228253. doi: 10.1371/journal.pone.0228253 (PMC6980647; doi:10.1371/journal.pone.0228253)
Supplement: S5 Fig — (PDF) [file pone.0228253.s005.pdf]

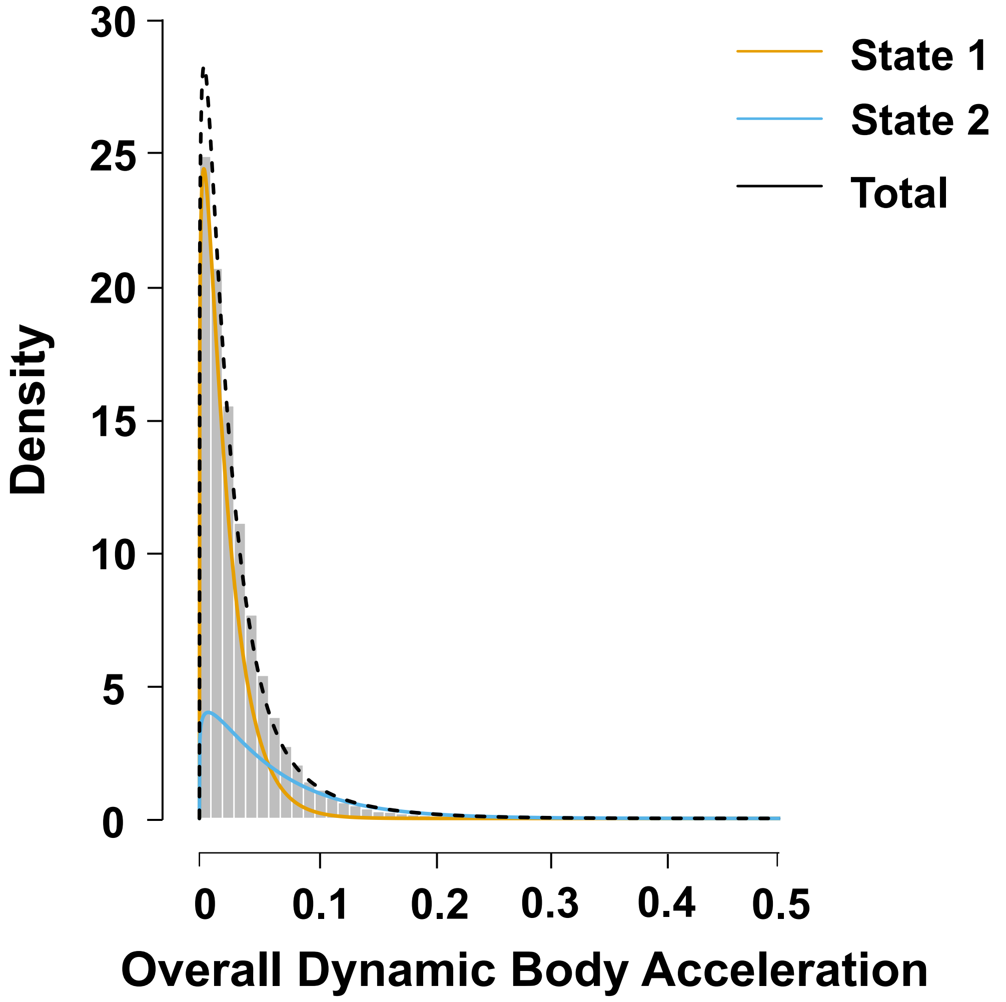

S5 Fig. State-dependent densities of overall dynamic body acceleration (ODBA) for sixgill sharks. Densities are weighted by the frequency of observations that correspond to each state. State 1 corresponds to relatively low levels of ODBA, while state 2 represents overall higher ODBA levels. The marginal density is the sum of the weighted-state dependent densities. ODBA values greater than 0.5 were truncated for clearer illustration and higher values were observed.
