# Supplementary material for: Diel patterns in swimming behavior of a vertically migrating deepwater shark, the bluntnose sixgill (Hexanchus griseus)
Source: PLoS One. 2020 Jan 24;15(1):e0228253. doi: 10.1371/journal.pone.0228253 (PMC6980647; doi:10.1371/journal.pone.0228253)
Supplement: S6 Fig — (PDF) [file pone.0228253.s006.pdf]

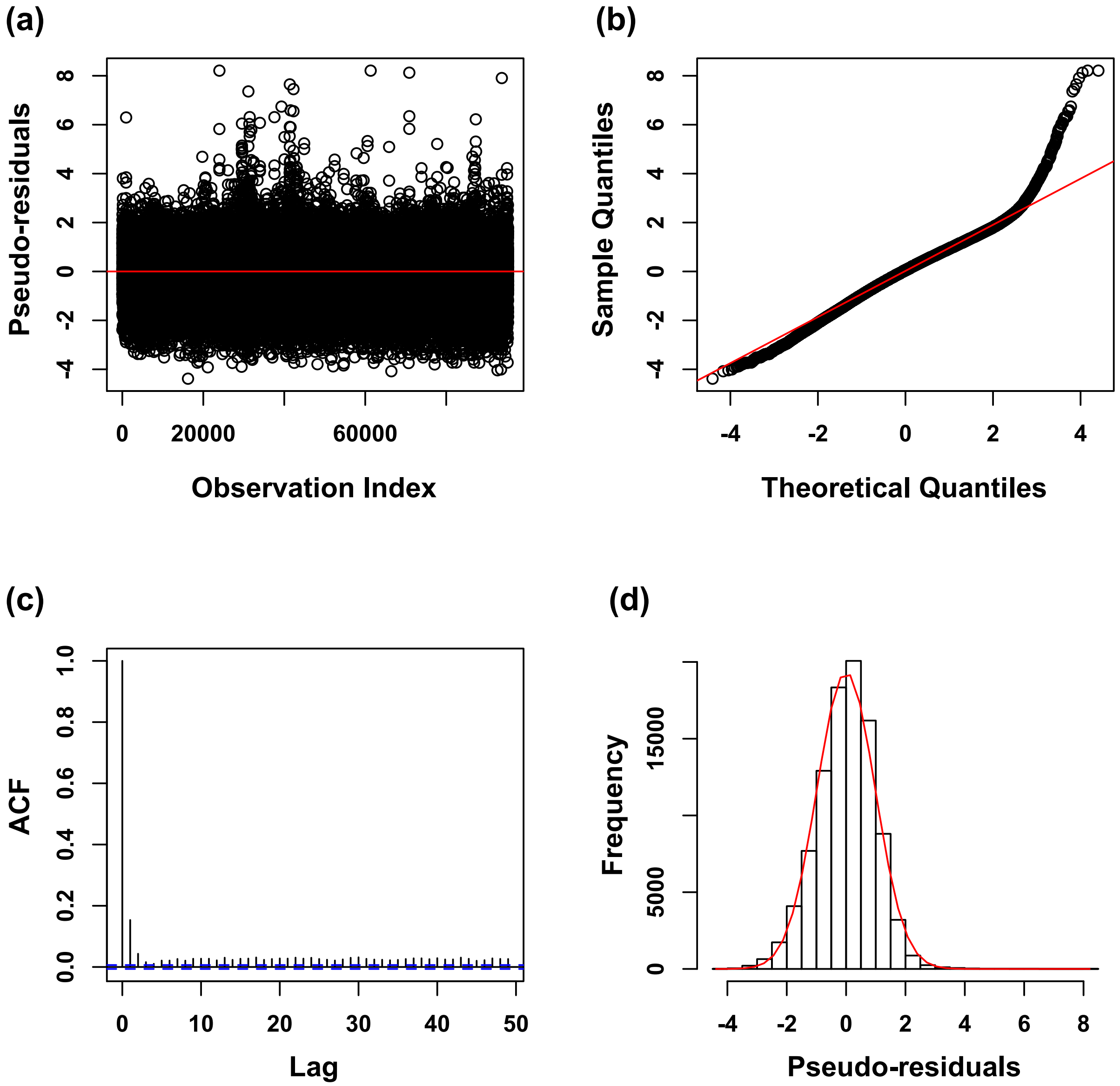

S6 Fig. Diagnostic plots for the two-state hidden Markov model with the best fit on overall dynamic body acceleration. (a) Time series of pseudo-residuals from the model with zero-centered line (red). (b) Quantile-quantile (Q-Q) plot for pseudo-residuals against the standard normal distribution with 1:1 line (red). (c) Pseudo-residual sample autocorrelation function. (d) Distribution of pseudo-residuals with fitted normal curve (red).
