# Supplementary material for: Diel patterns in swimming behavior of a vertically migrating deepwater shark, the bluntnose sixgill (Hexanchus griseus)
Source: PLoS One. 2020 Jan 24;15(1):e0228253. doi: 10.1371/journal.pone.0228253 (PMC6980647; doi:10.1371/journal.pone.0228253)
Supplement: S7 Fig — (PDF) [file pone.0228253.s007.pdf]

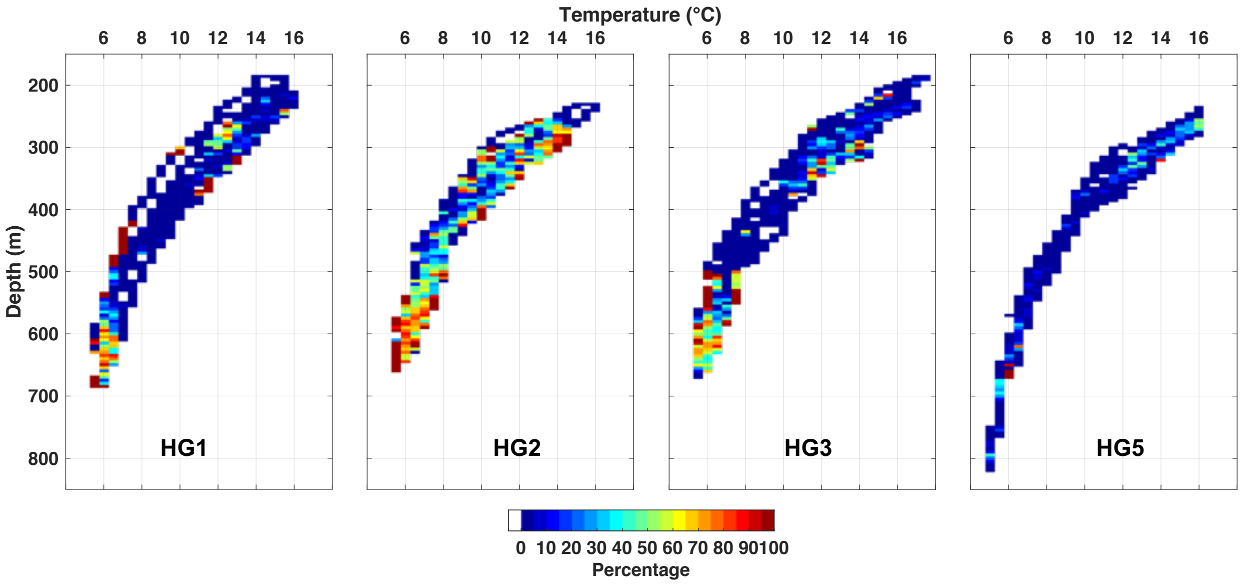

S7 Fig. Activity of sixgill sharks along a vertical profile of depth and ambient water temperature. Each grid cell (0.5 m × 0.5 °C) is color-coded by the percentage of observations corresponding to the high-activity state (state 2) from the decoded state sequence derived from the best-fit hidden Markov model.
