# Supplementary material for: Diel patterns in swimming behavior of a vertically migrating deepwater shark, the bluntnose sixgill (Hexanchus griseus)
Source: PLoS One. 2020 Jan 24;15(1):e0228253. doi: 10.1371/journal.pone.0228253 (PMC6980647; doi:10.1371/journal.pone.0228253)
Supplement: S8 Fig — (PDF) [file pone.0228253.s008.pdf]

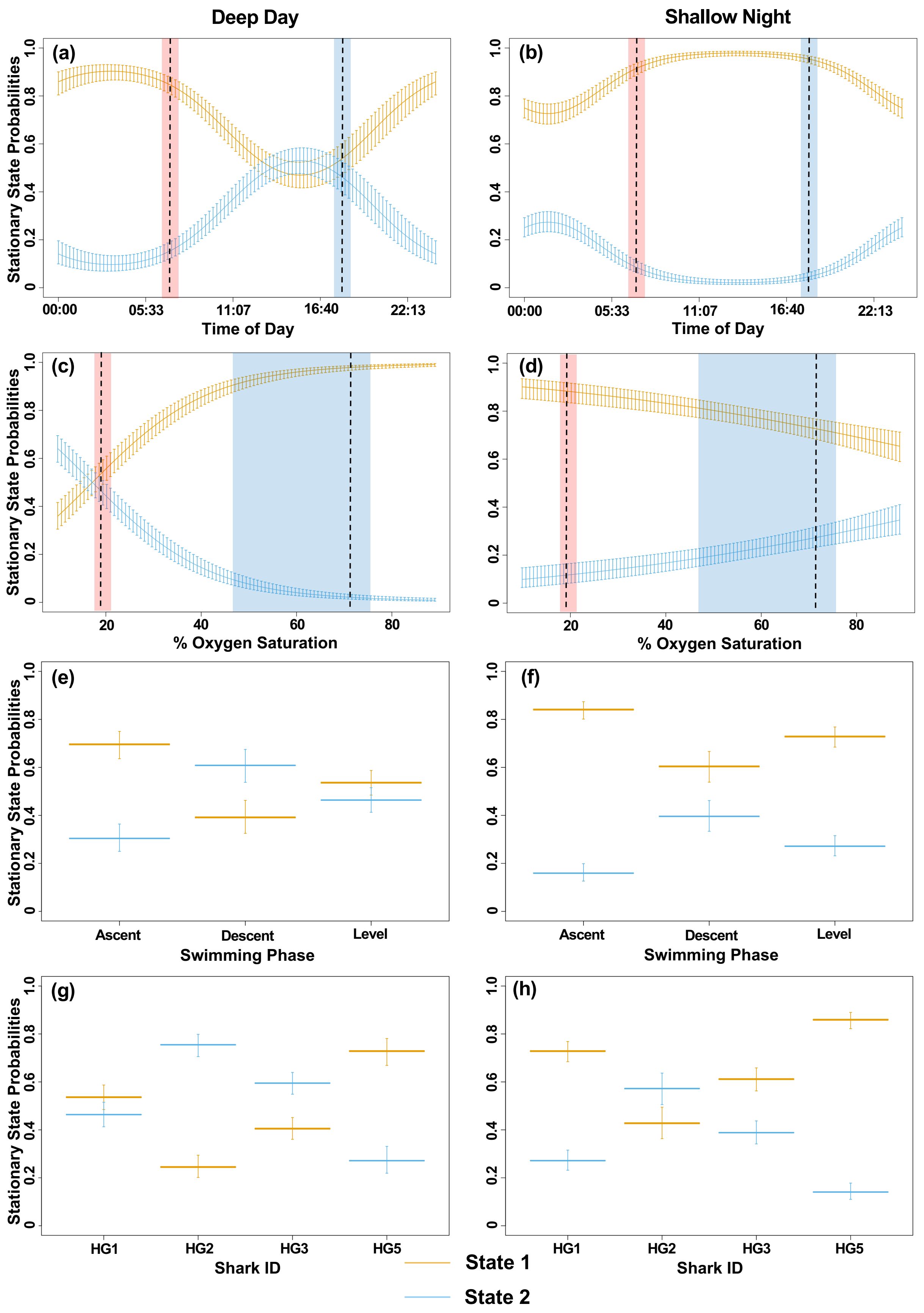

Deep Day
Shallow Night
(a)
(b)
(c)
(d)
(e)
(f)
(g)
(h)
Stationary State Probabilities
Time of Day
00:00
05:33
11:07
16:40
22:13
% Oxygen Saturation
20
40
60
80
Swimming Phase
Ascent
Descent
Level
Shark ID
HG1
HG2
HG3
HG5
0
0.2
0.4
0.6
0.8
1.0
State 1
State 2

S8 Fig. Stationary state probabilities from the best-fit hidden Markov model. Estimated stationary (equilibrium) state probabilities as a function of (a-b) time of day, (c-d) dissolved oxygen saturation, (e-f) swimming phase, and (g-h) shark ID from the best-fit hidden Markov model. Stationary state probabilities represent the marginal probability of a state at a fixed value of the covariate for deep day and shallow night diel phases. State 1 (orange) and state 2 (blue) correspond to relatively low and high levels of activity, respectively. Error bars represent 95% pointwise confidence limits. Median values (dashed lines) and interquartile ranges (shaded bars) are shown for the (a-b) timing of the end of dawn descent (red) and start of dusk ascent (blue) segments of vertical migrations and (c-d) dissolved oxygen saturations experienced during deep day (red) and shallow night (blue) diel phases.
