# Supplementary material for: Diel patterns in swimming behavior of a vertically migrating deepwater shark, the bluntnose sixgill (Hexanchus griseus)
Source: PLoS One. 2020 Jan 24;15(1):e0228253. doi: 10.1371/journal.pone.0228253 (PMC6980647; doi:10.1371/journal.pone.0228253)
Supplement: S9 Fig — (PDF) [file pone.0228253.s009.pdf]

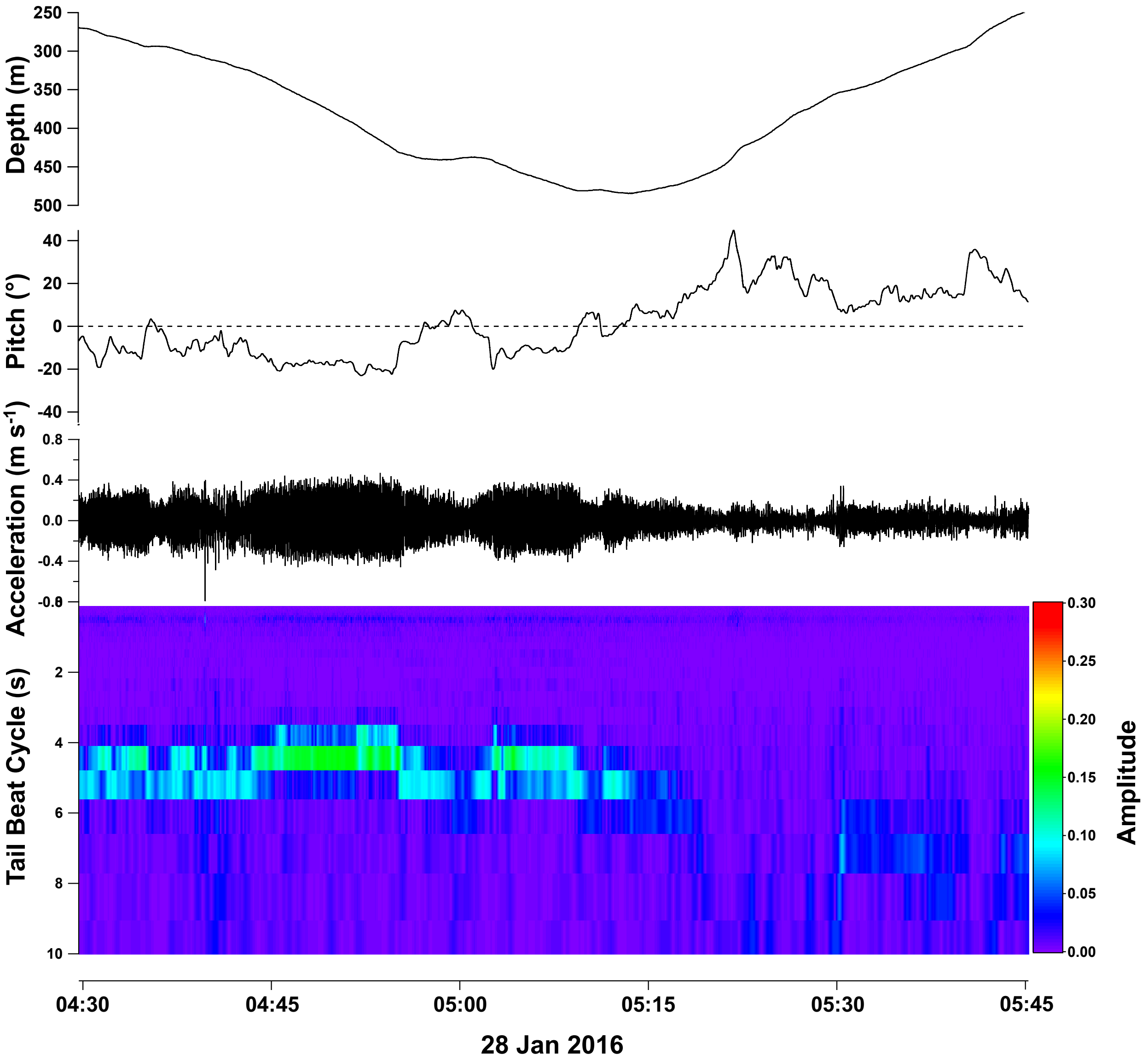

S9 Fig. Swimming performance during a vertical movement. Time series of depth, pitch, swaying acceleration caused by tail beat, and continuous wavelet spectrogram of swaying acceleration (color-coded by amplitude of acceleration) during a 75-minute vertical movement by HG3. Negative pitch values indicate the shark was oriented head-downward. From 05:15 through 05:45, a lack of strong signal in amplitude of acceleration indicates the shark was predominantly gliding uphill.
