# Supplementary material for: Diel patterns in swimming behavior of a vertically migrating deepwater shark, the bluntnose sixgill (Hexanchus griseus)
Source: PLoS One. 2020 Jan 24;15(1):e0228253. doi: 10.1371/journal.pone.0228253 (PMC6980647; doi:10.1371/journal.pone.0228253)
Supplement: S1 Table — (PDF) [file pone.0228253.s010.pdf]

S1 Table. Results of Kruskal-Wallis test examining the influence of diel depth phase on vertical displacement rate. A Dunn-Šidák correction was used for multiple comparisons due to unequal sample size among phases. Median values reported include interquartile range. Diel depth phases without a shared letter were significantly different.

Shark ID	Shallow Night ROM (m min^{-1})	Deep Day ROM (m min^{-1})	Dawn Descent ROM (m min^{-1})	Dusk Ascent ROM (m min^{-1})	chi-square	*P*
HG1	1.7 (0.8-3.0)^{a}	1.2 (0.5-2.6)^{b}	1.2 (0.5-3.4)^{b}	3.5 (1.8-6.2)^{c}	271.0556	<0.0001
HG2	2.6 (1.2-4.5)^{a}	2.0 (0.9-3.4)^{b}	4.1 (2.1-6.1)^{c}	2.0 (1.0-3.5)^{b}	167.3283	<0.0001
HG3	1.9 (0.9-3.4)^{a}	1.2 (0.5-2.3)^{b}	5.3 (3.3-7.4)^{c}	4.0 (2.1-7.0)^{d}	817.0743	<0.0001
HG4	3.2 (1.2-6.6)^{a}	1.7 (0.7-3.5)^{b}	7.1 (4.2-9.7)^{c}	4.7 (2.2-7.0)^{d}	412.4358	<0.0001
HG5	2.7 (1.5-4.0)^{a}	2.2 (1.2-3.5)^{b}	3.4 (1.8-4.7)^{c}	3.5 (2.6-4.5)^{c}	85.7596	<0.0001

ROM, rate of movement
