# Supplementary material for: Diel patterns in swimming behavior of a vertically migrating deepwater shark, the bluntnose sixgill (Hexanchus griseus)
Source: PLoS One. 2020 Jan 24;15(1):e0228253. doi: 10.1371/journal.pone.0228253 (PMC6980647; doi:10.1371/journal.pone.0228253)
Supplement: S2 Table — (PDF) [file pone.0228253.s011.pdf]

S2 Table. Results of Kruskal-Wallis test examining the influence of diel depth phase on pitch and acceleration metrics. A Dunn-Šidák correction was used for multiple comparisons due to unequal sample size among phases. Median values reported include interquartile range. Diel depth phases without a shared letter were significantly different.

	**Shark ID**	**HG1**	**HG2**	**HG3**	**HG5**
**Pitch (°)**	Shallow Night	-0.3 (-5.5 to 3.7)[a]	-1.5 (-8.8 to 6.8)[a]	-0.8 (-5.8 to 3.8)[a]	0.6 (-7.6 to 6.4)[a]
	Deep Day	1.0 (-2.8 to 3.9)[b]	0.2 (-6.0 to 6.9)[b]	1.3 (-2.2 to 4.7)[b]	2.9 (-6.1 to 7.2)[b]
	Dawn Descent	-1.5 (-6.6 to 1.2)[c]	-9.2 (-12.6 to -4.1)[c]	-11.8 (-14.8 to -8.0)[c]	-11.0 (-14.7 to -4.1)[c]
	Dusk Ascent	8.2 (3.0 to 18.4)[d]	5.0 (-0.0 to 9.0)[d]	7.7 (3.7-15.3)[d]	8.6 (6.5 to 11.0)[d]
	chi-square	362.8813	215.9827	810.0148	337.9918
	P	<0.0001	<0.0001	<0.0001	<0.0001
**Tail Beat Frequency (Hz)**	Shallow Night	0.20 (0.18-0.21)[a]	0.15 (0.13-0.16)[a]	0.21 (0.18-0.21)[a]	0.20 (0.16-0.27)[a]
	Deep Day	0.16 (0.15-0.18)[b]	0.13 (0.13-0.15)[b]	0.19 (0.18-0.21)[b]	0.18 (0.15-0.21)[b]
	Dawn Descent	0.15 (0.15-0.15)[c]	0.15 (0.13-0.16)[c]	0.24 (0.21-0.24)[c]	0.17 (0.15-0.18)[c]
	Dusk Ascent	0.18 (0.18-0.18)[a]	0.14 (0.13-0.15)[a]	0.18 (0.18-0.20)[d]	0.21 (0.19-0.22)[a]
	chi-square	998.8428	281.5264	625.3958	161.9906
	P	<0.0001	<0.0001	<0.0001	<0.0001
**Tail Beat Amplitude**	Shallow Night	0.06 (0.03-0.11)[a]	0.09 (0.07-0.12)[a]	0.05 (0.04-0.07)[a]	0.04 (0.03-0.06)[a]
	Deep Day	0.08 (0.06-0.10)[b]	0.11 (0.08-0.13)[b]	0.07 (0.06-0.09)[b]	0.04 (0.03-0.06)[a]
	Dawn Descent	0.13 (0.11-0.16)[c]	0.12 (0.08-0.15)[c]	0.08 (0.07-0.11)[c]	0.08 (0.05-0.10)[b]
	Dusk Ascent	0.07 (0.05-0.09)[a]	0.08 (0.07-0.10)[d]	0.05 (0.03-0.09)[a]	0.03 (0.02-0.04)[c]
	chi-square	741.6099	172.6446	704.4636	233.1664
	P	<0.0001	<0.0001	<0.0001	<0.0001
**ODBA ($m\ s^{-2}$)**	Shallow Night	0.13 (0.10-0.20)[a]	0.19 (0.15-0.23)[a]	0.17 (0.14-0.20)[a]	0.12 (0.10-0.16)[a]
	Deep Day	0.18 (0.16-0.20)[b]	0.23 (0.18-0.26)[b]	0.24 (0.20-0.30)[b]	0.19 (0.14-0.22)[a]
	Dawn Descent	0.22 (0.20-0.27)[c]	0.22 (0.19-0.26)[b]	0.22 (0.19-0.26)[c]	0.13 (0.10-0.16)[b]
	Dusk Ascent	0.16 (0.14-0.20)[b]	0.20 (0.17-0.23)[c]	0.17 (0.12-0.25)[d]	0.12 (0.11-0.14)[a]
	chi-square	686.6868	296.8927	1017.232	197.363
	P	<0.0001	<0.0001	<0.0001	<0.0001
