# Supplementary material for: Diel patterns in swimming behavior of a vertically migrating deepwater shark, the bluntnose sixgill (Hexanchus griseus)
Source: PLoS One. 2020 Jan 24;15(1):e0228253. doi: 10.1371/journal.pone.0228253 (PMC6980647; doi:10.1371/journal.pone.0228253)
Supplement: S3 Table — (PDF) [file pone.0228253.s012.pdf]

S3 Table. Principal component analysis (PCA) loadings for components (1-4). Loadings indicate the correlation between the component and each variable sub-sampled over 1-min means.

Variable	PC1	PC2	PC3	PC4
Depth (m)	0.513	0.310	0.536	0.595
Water Temperature (°C)	-0.518	-0.217	-0.253	0.788
Intramuscular Temperature (°C)	-0.453	0.889		
% Oxygen Saturation	-0.513	-0.257	0.805	-0.149
Proportion Variance Explained	0.891	0.085	0.015	0.009
