# Supplementary material for: Diel patterns in swimming behavior of a vertically migrating deepwater shark, the bluntnose sixgill (Hexanchus griseus)
Source: PLoS One. 2020 Jan 24;15(1):e0228253. doi: 10.1371/journal.pone.0228253 (PMC6980647; doi:10.1371/journal.pone.0228253)
Supplement: S4 Table — (PDF) [file pone.0228253.s013.pdf]

**S4 Table. Ranked generalized additive mixed models of effects on overall dynamic body acceleration.** Phase corresponds to swimming phase (ascent, descent, level). s() denotes a smooth term and t2() denotes a tensor product smooth (interaction). Values in bold indicate the best-fit model. n = 15890.

| Model | df | logLik | r2 (adj.) | $AIC_c$ | $\Delta AIC_c$ | *w* | %DE |
|---|---|---|---|---|---|---|---|
| **Phase + s(Time of Day) + s(PC1)** | **9** | **-2428.5** | **0.206** | **4875.0** | **0.0** | **1.00** | **20.7** |
| Phase + s(Time of Day) + s(Depth, m) | 9 | -2438.5 | 0.153 | 4895.0 | 20.0 | $4.50 \times 10^{-5}$ | 15.4 |
| Phase + s(Time of Day) + s(Water Temperature, °C) | 9 | -2438.9 | 0.178 | 4895.8 | 20.8 | $2.99 \times 10^{-5}$ | 17.9 |
| Phase + s(PC1) | 8 | -2441.0 | 0.178 | 4897.9 | 23.0 | $1.02 \times 10^{-5}$ | 17.8 |
| Phase + s(Water Temperature, °C) | 8 | -2454.7 | 0.153 | 4925.4 | 50.4 | $1.14 \times 10^{-11}$ | 15.4 |
| Phase + s(Time of Day) + s(Intramuscular Temperature, °C) | 9 | -2454.3 | 0.157 | 4926.6 | 51.6 | $6.26 \times 10^{-12}$ | 15.7 |
| Phase + t2(Time of Day × Water Temperature, °C) | 11 | -2456.4 | 0.146 | 4934.7 | 59.8 | $1.05 \times 10^{-13}$ | 14.7 |
| Phase + t2(Time of Day × PC1) | 11 | -2462.0 | 0.150 | 4946.0 | 71.0 | $3.73 \times 10^{-16}$ | 15.0 |
| Phase + t2(Time of Day × Depth, m) | 11 | -2473.3 | 0.108 | 4968.6 | 93.6 | $4.72 \times 10^{-21}$ | 10.9 |
| Phase + t2(Time of Day × Intramuscular Temperature, °C) | 11 | -2474.5 | 0.148 | 4971.0 | 96.0 | $1.40 \times 10^{-21}$ | 14.9 |
| Phase + t2(Time of Day × % Oxygen Saturation) | 11 | -2475.1 | 0.106 | 4972.2 | 97.2 | $7.67 \times 10^{-22}$ | 10.6 |
| Phase + s(Time of Day) + s(% Oxygen Saturation) | 9 | -2477.9 | 0.095 | 4973.8 | 98.9 | $3.41 \times 10^{-22}$ | 9.6 |
| Phase + s(Time of Day) | 7 | -2481.2 | 0.092 | 4976.4 | 101.5 | $9.31 \times 10^{-23}$ | 9.3 |
| Phase + s(Intramuscular Temperature, °C) | 8 | -2482.9 | 0.122 | 4981.7 | 106.8 | $6.51 \times 10^{-24}$ | 12.3 |
| Phase + s(Depth, m) | 8 | -2492.7 | 0.076 | 5001.4 | 126.4 | $3.51 \times 10^{-28}$ | 7.5 |
| Phase + s(% Oxygen Saturation) | 8 | -2506.1 | 0.086 | 5028.2 | 153.2 | $5.38 \times 10^{-34}$ | 8.5 |
| Phase | 6 | -2521.5 | 0.042 | 5055.0 | 180.1 | $7.87 \times 10^{-40}$ | 4.2 |
| s(Time of Day) + s(PC1) | 7 | -2916.6 | 0.150 | 5847.2 | 972.3 | $7.47 \times 10^{-212}$ | 15.1 |
| t2(Time of Day × Depth, m) | 9 | -2920.5 | 0.095 | 5859.0 | 984.1 | $2.05 \times 10^{-214}$ | 9.5 |
| s(Time of Day) + s(Depth, m) | 7 | -2923.8 | 0.098 | 5861.7 | 986.7 | $5.50 \times 10^{-215}$ | 9.9 |
| s(Time of Day) + s(Water Temperature, °C) | 7 | -2930.2 | 0.120 | 5874.4 | 999.4 | $9.52 \times 10^{-218}$ | 12.1 |
| s(PC1) | 6 | -2934.2 | 0.118 | 5880.4 | 1005.4 | $4.70 \times 10^{-219}$ | 11.8 |
| s(Time of Day) + s(Intramuscular Temperature, °C) | 7 | -2940.8 | 0.103 | 5895.5 | 1020.6 | $2.46 \times 10^{-222}$ | 10.4 |
| t2(Time of Day × Water Temperature, °C) | 9 | -2941.4 | 0.089 | 5900.8 | 1025.8 | $1.79 \times 10^{-223}$ | 8.9 |
| s(Water Temperature, °C) | 6 | -2944.9 | 0.093 | 5901.7 | 1026.8 | $1.10 \times 10^{-223}$ | 9.3 |
| t2(Time of Day × % Oxygen Saturation) | 9 | -2942.6 | 0.054 | 5903.2 | 1028.3 | $5.21 \times 10^{-224}$ | 5.4 |
| s(Time of Day) | 5 | -2947.8 | 0.046 | 5905.5 | 1030.6 | $1.66 \times 10^{-224}$ | 4.6 |

| **Model** | **df** | **logLik** | **r2 (adj.)** | **AIC$_c$** | **ΔAIC$_c$** | ***w*** | **%DE** |
|---|---|---|---|---|---|---|---|
| t2(Time of Day × Intramuscular Temperature, °C) | 9 | -2947.1 | 0.098 | 5912.3 | 1037.3 | $5.60 \times 10^{-226}$ | 9.8 |
| s(Time of Day) + s(% Oxygen Saturation) | 7 | -2949.7 | 0.048 | 5913.4 | 1038.4 | $3.23 \times 10^{-226}$ | 4.8 |
| t2(Time of Day × PC1) | 9 | -2949.8 | 0.097 | 5917.7 | 1042.7 | $3.82 \times 10^{-227}$ | 9.7 |
| s(Depth, m) | 6 | -2968.0 | 0.027 | 5948.1 | 1073.1 | $9.43 \times 10^{-234}$ | 2.6 |
| s(Intramuscular Temperature, °C) | 6 | -2969.5 | 0.069 | 5951.1 | 1076.1 | $2.11 \times 10^{-234}$ | 6.9 |
| Intercept only | 4 | -2976.9 | | 5961.8 | 1086.9 | $9.72 \times 10^{-237}$ | |
| s(% Oxygen Saturation) | 6 | -2976.8 | 0.039 | 5965.6 | 1090.6 | $1.52 \times 10^{-237}$ | 3.8 |

df, degrees of freedom; logLik, maximum log-likelihood; %DE, percent deviance explained
