# Supplementary material for: Diel patterns in swimming behavior of a vertically migrating deepwater shark, the bluntnose sixgill (Hexanchus griseus)
Source: PLoS One. 2020 Jan 24;15(1):e0228253. doi: 10.1371/journal.pone.0228253 (PMC6980647; doi:10.1371/journal.pone.0228253)
Supplement: S5 Table — (PDF) [file pone.0228253.s014.pdf]

S5 Table. Results from the best generalized additive mixed models fitted to overall dynamic body acceleration (ODBA). Phase corresponds to swimming phase (ascent, descent, level) and reference level is set to ascent for estimation of parametric coefficients. s() denotes a smoother term. n = 15890.

Model and terms	df	Estimate ± SE	t-value	edf	F-ratio	*P*
ODBA ~ Phase + s(Time of Day) + s(PC1)						
Phase	2				540.1	< 0.0001
Intercept		-1.737 ± 0.063	-27.477			< 0.0001
Descent		0.177 ± 0.006	29.023			< 0.0001
Level		-0.032 ± 0.005	-6.219			< 0.0001
s(Time of Day)				7.068	19.66	< 0.0001
s(PC1)				8.869	44.06	< 0.0001
ODBA ~ Phase + s(Time of Day) + s(Depth, m)						
Phase	2				542.5	< 0.0001
Intercept		-1.735 ± 0.069	-25.245			< 0.0001
Descent		0.177 ± 0.006	29.064			< 0.0001
Level		-0.034 ± 0.005	-6.446			< 0.0001
s(Time of Day)				6.093	9.329	< 0.0001
s(Depth, m)				7.796	23.043	
ODBA ~ Phase + s(Time of Day) + s(Water Temperature, °C)						
Phase	2				555.6	< 0.0001
Intercept		-1.738 ± 0.069	-25.202			< 0.0001
Descent		0.180 ± 0.006	29.480			< 0.0001
Level		-0.033 ± 0.005	-6.308			< 0.0001
s(Time of Day)				7.138	17.86	< 0.0001
s(Water Temperature, °C)				8.738	37.82	< 0.0001

df, degrees of freedom; SE, standard error; edf, estimated degrees of freedom
