# Supplementary material for: Diel patterns in swimming behavior of a vertically migrating deepwater shark, the bluntnose sixgill (Hexanchus griseus)
Source: PLoS One. 2020 Jan 24;15(1):e0228253. doi: 10.1371/journal.pone.0228253 (PMC6980647; doi:10.1371/journal.pone.0228253)
Supplement: S6 Table — (PDF) [file pone.0228253.s015.pdf]

S6 Table. Ranked hidden Markov models of effects on activity states based on overall dynamic body acceleration. Values in bold indicate the best-fit model. Phase corresponds to swimming phase (ascent, descent, level). n = 95300.

Model	logLik	AIC	ΔAIC	*w*
**ID, Phase, Time of Day, % Oxygen Saturation, Time of Day × % Oxygen Saturation**	**233964.0**	**-467874.0**	**0.0**	**0.843**
ID, Phase, Time of Day, PC1, Time of Day × PC1	233962.3	-467870.6	3.4	0.157
ID, Phase, Time of Day, Intramuscular Temperature (°C), Time of Day × Intramuscular Temperature (°C)	233951.1	-467848.1	25.9	2.05×10^{-6}
ID, Phase, Time of Day, Water Temperature (°C), Time of Day × Water Temperature (°C)	233940.7	-467827.3	46.7	6.33×10^{-11}
ID, Time of Day, % Oxygen Saturation, Time of Day × % Oxygen Saturation	233919.3	-467792.6	81.4	1.77×10^{-18}
ID, Time of Day, PC1, Time of Day × PC1	233910.2	-467774.5	99.5	2.09×10^{-22}
ID, Phase, Time of Day, Depth (m), Time of Day × Depth (m)	233912.9	-467771.9	102.1	5.78×10^{-23}
ID, Time of Day, Water Temperature (°C), Time of Day × Water Temperature (°C)	233891.6	-467737.2	136.8	1.66×10^{-30}
ID, Time of Day, Intramuscular Temperature (°C), Time of Day × Intramuscular Temperature (°C)	233888.6	-467731.1	142.9	8.17×10^{-32}
ID, Time of Day, Depth (m), Time of Day × Depth (m)	233876.2	-467706.3	167.7	3.33×10^{-37}
ID, Phase, Time of Day, Depth (m)	233848.8	-467651.6	222.4	4.26×10^{-49}
ID, Phase, Time of Day, PC1	233846.5	-467646.9	227.1	4.21×10^{-50}
ID, Phase, Time of Day, Intramuscular Temperature (°C)	233846.0	-467646.0	228.0	2.64×10^{-50}
ID, Phase, Time of Day, Water Temperature (°C)	233842.6	-467639.3	234.7	9.21×10^{-52}
Phase, Time of Day, Intramuscular Temperature (°C), Time of Day × Intramuscular Temperature (°C)	233840.7	-467639.3	234.7	9.47×10^{-52}
ID, Phase, Time of Day, % Oxygen Saturation	233838.3	-467630.6	243.4	1.17×10^{-53}
ID, Phase, Time of Day	233821.6	-467601.1	272.9	4.73×10^{-60}
Phase, Time of Day, PC1, Time of Day × PC1	233817.4	-467592.7	281.3	7.08×10^{-62}
Phase, Time of Day, % Oxygen Saturation, Time of Day × % Oxygen Saturation	233816.2	-467590.4	283.6	2.29×10^{-62}
ID, Time of Day, Depth (m)	233800.4	-467562.8	311.2	2.32×10^{-68}
ID, Time of Day, Intramuscular Temperature (°C)	233797.4	-467556.8	317.2	1.16×10^{-69}
ID, Time of Day, PC1	233795.9	-467553.8	320.2	2.49×10^{-70}
ID, Time of Day, Water Temperature (°C)	233794.1	-467550.2	323.8	4.09×10^{-71}
ID, Time of Day, % Oxygen Saturation	233793.6	-467549.3	324.7	2.63×10^{-71}
ID, Time of Day	233785.1	-467536.2	337.8	3.80×10^{-74}
ID, Depth (m)	233781.4	-467532.8	341.2	6.99×10^{-75}

**Model**	**logLik**	**AIC**	**ΔAIC**	***w***
ID, PC1	233777.2	-467524.4	349.6	1.02×10^{-76}
Phase, Time of Day, Water Temperature (°C), Time of Day × Water Temperature (°C)	233783.1	-467524.1	349.9	9.09×10^{-77}
ID, % Oxygen Saturation	233771.4	-467512.8	361.2	3.18×10^{-79}
ID, Intramuscular Temperature (°C)	233771.2	-467512.4	361.6	2.57×10^{-79}
ID, Water Temperature (°C)	233766.3	-467502.6	371.4	1.90×10^{-81}
Time of Day, % Oxygen Saturation, Time of Day × % Oxygen Saturation	233740.9	-467447.8	426.2	2.46×10^{-93}
Phase, Time of Day, Depth (m), Time of Day × Depth (m)	233744.4	-467446.7	427.3	1.43×10^{-93}
ID, Phase	233738.7	-467443.4	430.6	2.69×10^{-94}
Time of Day, Intramuscular Temperature (°C), Time of Day × Intramuscular Temperature (°C)	233735.4	-467436.8	437.2	9.77×10^{-96}
Phase, Time of Day, % Oxygen Saturation	233734.1	-467434.3	439.7	2.78×10^{-96}
Phase, Time of Day, PC1	233732.5	-467431.0	443.0	5.59×10^{-97}
Phase, Time of Day, Intramuscular Temperature (°C)	233732.1	-467430.2	443.8	3.74×10^{-97}
Time of Day, PC1, Time of Day × PC1	233730.3	-467426.7	447.3	6.32×10^{-98}
ID	233711.6	-467397.2	476.8	2.53×10^{-104}
Phase, Time of Day, Water Temperature (°C)	233714.0	-467394.1	479.9	5.28×10^{-105}
Phase, Time of Day, Depth (m)	233703.1	-467372.1	501.9	9.05×10^{-110}
Time of Day, Water Temperature (°C), Time of Day × Water Temperature (°C)	233702.4	-467370.8	503.2	4.50×10^{-110}
Time of Day, Depth (m), Time of Day x Depth (m)	233678.4	-467322.9	551.1	1.82×10^{-120}
Phase, Time of Day	233669.7	-467309.5	564.5	2.25×10^{-123}
Time of Day, % Oxygen Saturation	233664.7	-467303.4	570.6	1.08×10^{-124}
% Oxygen Saturation	233650.5	-467283.0	591.0	3.93×10^{-129}
Time of Day, PC1	233653.5	-467281.0	593.0	1.48×10^{-129}
Intramuscular Temperature (°C)	233647.7	-467277.3	596.7	2.32×10^{-130}
Time of Day, Intramuscular Temperature (°C)	233648.3	-467270.5	603.5	7.74×10^{-132}
PC1	233644.2	-467270.3	603.7	7.05×10^{-132}
Time of Day, Water Temperature (°C)	233639.8	-467253.6	620.4	1.65×10^{-135}
Time of Day, Depth (m)	233638.6	-467251.3	622.7	5.15×10^{-136}
Water Temperature (°C)	233627.4	-467236.7	637.3	3.54×10^{-139}
Depth (m)	233625.8	-467233.7	640.3	7.69×10^{-140}
Time of Day	233619.6	-467217.3	656.7	2.14×10^{-143}

Model	logLik	AIC	ΔAIC	*w*
Phase	233578.8	-467135.6	738.4	3.90×10^{-161}
No covariates	233544.2	-467074.4	799.6	2.04×10^{-174}

logLik, maximum log-likelihood; ID, individual shark
